# Chemical Constituent of β-Glucuronidase Inhibitors from the Root of *Neolitsea acuminatissima*

**DOI:** 10.3390/molecules25215170

**Published:** 2020-11-06

**Authors:** Chu-Hung Lin, Hsiao-Jung Chou, Chih-Chi Chang, Ih-Sheng Chen, Hsun-Shuo Chang, Tian-Lu Cheng, Yueh-Hsiung Kuo, Horng-Huey Ko

**Affiliations:** 1 Botanical Drug Technology Division, Biomedical Technology and Device Research Laboratories, Industrial Technology Research Institute, Hsinchu 30011, Taiwan; chuhung.lin@gmail.com; 2 Department of Fragrance and Cosmetic Science, College of Pharmacy, Kaohsiung Medical University, Kaohsiung 80708, Taiwan; cc032324@gmail.com (H.-J.C.); newheart1920@hotmail.com (C.-C.C.); 3 School of Pharmacy, College of Pharmacy, Kaohsiung Medical University, Kaohsiung 80708, Taiwan; m635013@kmu.edu.tw (I.-S.C.); hschang@kmu.edu.tw (H.-S.C.); 4 Drug Development and Value Creation Center, Kaohsiung Medical University, Kaohsiung 80708, Taiwan; tlcheng@kmu.edu.tw; 5 Department of Biomedical Science and Environmental Biology, Kaohsiung Medical University, Kaohsiung 80708, Taiwan; 6 Department of Chinese Pharmaceutical Sciences and Chinese Medicine Resources, China Medical University, Taichung 40402, Taiwan; 7 Chinese Medicine Research Center, China Medical University, Taichung 40402, Taiwan; 8 Department of Biotechnology, Asia University, Taichung 41354, Taiwan

**Keywords:** *Neolitsea acuminatissima*, Lauraceae, root, carboline, eudesmanolide, β-Glucuronidase, chemotherapy-induced diarrhea (CID)

## Abstract

*Neolitsea acuminatissima* (Lauraceae) is an endemic plant in Taiwan. One new carboline alkaloid, demethoxydaibucarboline A (**1**), two new eudesmanolide-type sesquiterpenes, methyl-neolitacumone A (**2**), neolitacumone E (**3**), and twelve known compounds (**4**–**15**) were isolated from the root of *Neolitsea acuminatissima.* Their structures were elucidated by spectroscopic analysis. Glucuronidation represents a major metabolism process of detoxification for carcinogens in the liver. However, intestinal bacterial β-Glucuronidase (βG) has been considered pivotal to colorectal carcinogenesis. To develop specific bacterial-βG inhibitors with no effect on human βG, methanolic extract of roots of *N. acuminatissima* was selected to evaluate their anti-βG activity. Among the isolates, demethoxydaibucarboline A (**1**) and quercetin (**8**) showed a strong bacterial βG inhibitory effect with an inhibition ratio of about 80%. Methylneolitacumone A (**2**) and epicatechin (**10**) exhibited a moderate or weak inhibitory effect and the enzyme activity was less than 45% and 74%, respectively. These four compounds specifically inhibit bacterial βG but not human βG. Thus, they are expected to be used for the purpose of reducing chemotherapy-induced diarrhea (CID). The results suggest that the constituents of *N. acuminatissima* have the potential to be used as CID relief candidates. However, further investigation is required to determine their mechanisms of action.

## 1. Introduction

The *Neolitsea* genus (Lauraceae) is an important component with approximately 85 species and includes evergreen shrubs and small trees in the tropical and subtropical region of Asia [1]. Some plants of the genus *Neolitsea* have been used as folk/herbal medicine, such as treatment of rheumatic arthralgia, furuncle and carbuncle, and edema [2]. Moreover, crude extracts and some pure chemical constituents of the *Neolitsea* species exhibited antioxidant, antiaging, antimicrobial [2,3], anti-inflammation [4,5,6], tyrosinase inhibition [7], and antitumor activity [8,9]. Phytochemical studies of *Neolitsea* have revealed the presence of alkaloids, benzenoids, flavonoids, lignans, quinone, sesquiterpenes, steroids, terpenoids, and others [2,4,5,6,7,8,9,10]. This success has spurred the continuing search for more bioactive constituents of Formosan *Neolitsea* plants. *Neolitsea acuminatissima* (Hayata) Kanehira & Sasaki is an endemic species and evergreen small tree distributed in broad-leaved forests at high altitude throughout Taiwan [11]. An alkaloid, (+)-laurotetanine, of the stem bark of this plant was first published in 1965 by Tomita et al. [12]. Twenty compounds have further been isolated from the stem bark and among them, eudesmanolide sesquiterpenes, such as neolitacumones B and C, are known to have cytotoxicity against Hep 2,2,15 cells [8]. However, as study of the root of *N. acuminatissima* has not been performed, an investigation was carried out to search for additional valuable bioactivity from *N. acuminatissima* roots.

Irinotecan (CPT-11), a first-line chemotherapeutic agent, is essential for treating malignancies, such as brain, lung, colorectal, and pancreatic cancers [13]. One of the major dose-limiting toxicities of CPT-11 regimen is unpredictable and causes severe diarrhea: more than 80% of patients suffer from delayed-onset diarrhea, and with 30% to 40% of them having severe diarrhea (grade 3 to 5) [14,15]. This toxicity significantly affects quality of life and may threaten the success of cancer chemotherapy, thus resulting in decreasing the drug dose or even discontinuation of treatment. Therefore, treating CPT-11-induced diarrhea is a significant clinical need [16,17]. Microflora in the intestine play a pivotal role in CPT-11-induced diarrhea. As a prodrug, CPT-11 is converted by carboxylesterase to SN-38, the active metabolite responsible for both toxicity and antitumor activity [18]. SN-38 is further catalyzed to inactive SN-38 glucuronide (SN-38G) by UDP-glucuronosyltransferase in the liver and excreted into the bile with other major components, CPT-11 and SN-38 by P-glycoprotein [19]. However, bacterial β-Glucuronidase (βG) enzymes in intestinal microflora, such as *Escherichia coli*, may reconvert nontoxic SN-38G to toxic metabolite SN-38 and lead to damage of intestine epithelia cells and cause severe diarrhea [18,19]. Therefore, inhibiting intestinal *E. coli* βG (eβG) activity is expected to protect the intestines from injury and thus alleviate chemotherapy-induced diarrhea (CID), even enhancing the therapeutic index. Despite some antidiarrheal agents already used to treat CID clinically [14,15], these approaches have several drawbacks. Thus, development of eβG inhibitors from natural products are valuable and expected to mitigate CID.

In a preliminary anti-eβG screening assay, sixty-five species of Formosan lauraceous plants were selected to evaluate their anti-eβG activity. Among them, the methanolic extract of the root of *Neolitsea acuminatissima* (NARM) showed a strong inhibitory effect on eβG with an inhibition ratio of 68%, without affecting the activity of human βG (hβG). (The details are shown in the Supporting Information, Figure S1.) According to preliminary anti-eβG data, we proposed the existence of active constituents in *N. acuminatissima*, since the chemical constituents and biological activity of roots of this plant have seldom been investigated previously. It is worthy to verify the phytochemistry and medicinal treatment of NARM. Thus, NARM was selected as the candidate to investigate secondary metabolites for their anti-eβG activity. Here in this article, the structure elucidation of three new compounds and results of anti-eβG activity are reported.

## 2. Results and Discussion

### 2.1. Structure Elucidation of Compounds ***1**–**3***

**Figure 1:**
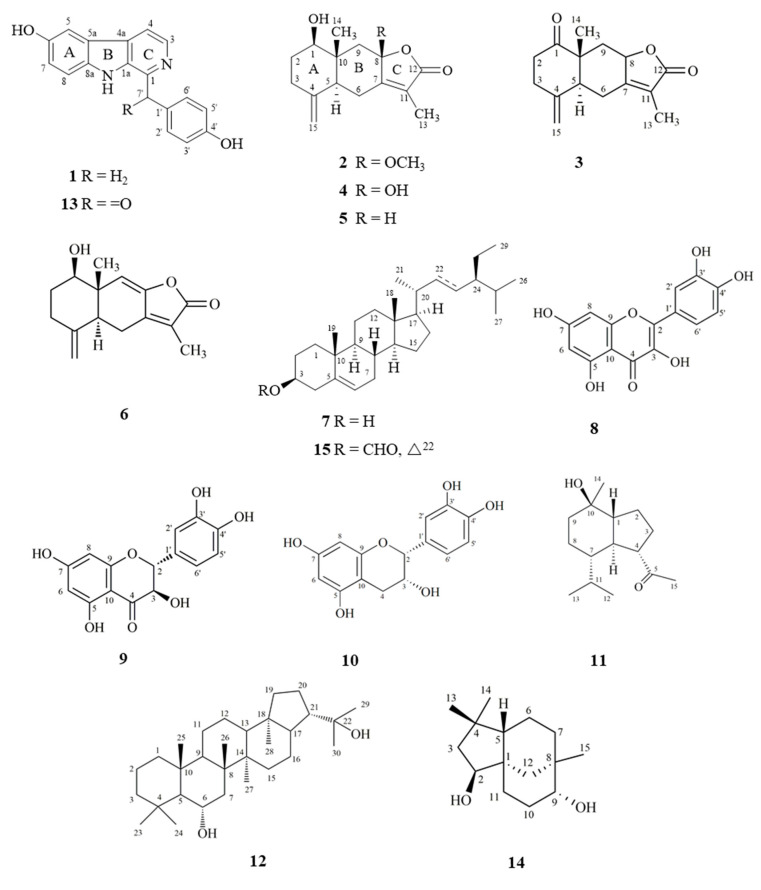
Structures of compounds (**1**–**15**) isolated from the root of *Neolitsea acuminatissima.*

Fifteen compounds (**1**–**15**), including three new compounds, were isolated from dichloromethane (NARD) and ethyl acetate (NARE) soluble layers of NARM. Their structures (Figure 1) were spectroscopically determined by FTIR and 1D and 2D NMR, and through comparison with those of reported analogs. The structure determination of three new compounds was described in the present paper.

Compound **1** was isolated as a yellowish powder. The molecular formula C_18_H_14_N_2_O_2_ was determined by ESIMS and HR-ESIMS (291.1127 [M + H]^+^). The IR spectrum exhibited absorption peaks attributable to a hydroxy group (3277 cm^−1^) and an aromatic ring (1610, 1583, and 1512 cm^−1^).

**Figure 2:**
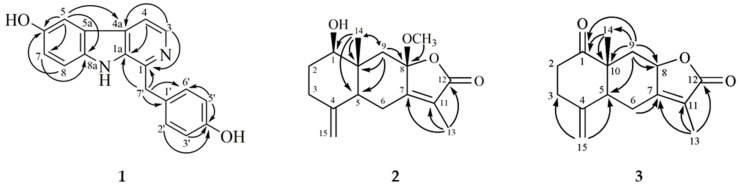
Key HMBC (H→C) correlations of **1**–**3**.

The UV spectrum of **1** was similar to that of daibucarboline A [4] and cecilin [20] and was consistent with a carboline moiety [20]. The ^1^H-NMR spectrum of **1** (Table 1) showed a trisubstituted aromatic ABX system (δ 7.10 (1H, dd, *J* = 8.8, 2.4 Hz, H-7), 7.40 (1H, d, *J* = 8.8 Hz, H-8), 7.56 (1H, d, *J* = 2.4 Hz, H-5)) on ring A, an NH proton (δ 10.32 (1H, br s)) on ring B, a pair of mutually coupled protons (δ 7.82 (1H, d, *J* = 5.2 Hz, H-4) and 8.24 (1H, d, *J* = 5.2 Hz, H-3)), and a *p*-hydroxybenzyl group (δ 4.37 (2H, s, H-7′), 6.70 (2H, d, *J* = 8.8 Hz, H-3′ and H-5′), and 7.20 (2H, d, *J* = 8.8 Hz, H-2′ and H-6′)) on ring C. The HMBC correlations (Figure 2) from H-7′ to C-1, C-1a, and C-1′ further confirmed the *p*-hydroxybenzyl group located at C-1 on ring C. A comparison of ^1^H and ^13^C data of **1** with those of cecilin [20] suggested that their structures are closely related, except that the hydroxy group of **1** replaced the methoxy group at C-6 of cecilin. This was supported by HMBC correlation from H-5 to C-6, H-8 to C-6, and the ESIMS of **1** contained less 14 amu [CH_2_] than cecilin. Meanwhile, the NMR spectra of **1** closely resembled those of daibucarboline A [4], except for the absence of a methoxy substituent at C-3 position. From these data, the structure of **1** was determined to be demethoxydaibucarboline A, which was further confirmed by HSQC, COSY, NOESY, and HMBC experiments.

Compound **2** was obtained as colorless needles with [α]${}_{D}^{25}$ +148.6° (*c* 0.27, CHCl_3_). The molecular formula C_16_H_22_O_4_ was deduced from the HRESIMS ion peak at *m/z* 301.1409 [M + Na]^+^ (calcd for C_16_H_22_O_4_Na 301.1410) with six degrees of unsaturation. Its IR spectrum exhibited absorption peaks corresponding to a hydroxy group (3419 cm^−1^) and an α,β-unsaturated lactone functional group (1733 cm^−1^). The UV spectrum showed absorbance maxima at 219 nm. The ^1^H-NMR spectrum (Table 1) exhibited signals for a tertiary methyl (δ 0.94 (3H, s, H-14)), a vinylic methyl (δ 1.86 (3H, d, *J* = 1.6 Hz, H-13)), and two terminal methylene protons (δ 4.64 (1H, d, *J* = 1.6 Hz) and 4.90 (1H, d, *J* = 1.6 Hz)), which are similar to those of known eudesmanolides, 1β-acetoxy-8β-hydroxyeudesman-4(15),7(11)-dien-8α,12-olide [21]. The ^13^C-NMR spectrum and DEPT experiments displayed 15 carbon signals that correspond to an eudesmanolide containing a conjugated γ-lactone carbonyl (δ 171.7), a terminal double bond (δ 108.1), a set of tetrasubstituted double bond (C-7 and -11), an acetal (δ 106.2, C-8), two methyls (C-13 and -14), four methylenes (C-2, -3, -6, and -9), two methines (C-1 and -5), and two quaternary carbons (C-4 and -10), respectively. The signals at δ 8.3 (C-13), 106.2 (C-8), 124.3 (C-11), 158.8 (C-7), and 171.7 (C-12) are characteristic of a 5-oxygenated-3-methyl-5-hydrofuran-2-one functional moiety of eudesmanolides [21]. The ^1^H and ^13^C-NMR data of **2** were similar to those of neolitacumone A (**5**) [8], except **2** has a methoxy group instead of a hydroxy group at C-8 of neolitacumone A (**5**), and the ESIMS of **2** had greater 14 amu than **5**. In addition, the location of the methoxy group of **2** at C-8 was further confirmed by HMBC correlation from OCH_3_-8 to C-8. The HMBC correlations from H-1 to C-2 (δ 30.8), C-9 (δ 46.4), and C-10 (δ 41.1); from H-13 to C-7 (δ 158.8), C-11 (δ 124.3), and C-12; from H-14 to C-1 (δ 78.8), C-5 (δ 49.6), C-9, and C-10; and from H-15a and H-15b to C-3 (δ 33.6) and C-5 further confirmed the planar structure of **2**. In addition, the *trans*-A/B ring junction of **2** was confirmed by observation of NOESY cross-peaks between δ 0.94 (H-14)/1.57 (H-2β), 0.94 (H-14)/2.47 (H-6β), and 2.78 (H-9β) and between δ 1.57 (H-2β)/2.31 (H-3β) [8]. Thus, the stereochemistry of **2** was confirmed as 1β-OH, 5α-H and 10β-CH_3_. In addition, NOESY correlations between δ 3.16 (8-OCH_3_)/0.94 (H-14) and 2.78 (H-9β) suggested a β-orientation of the methoxy group at C-8. From the above data, the structure of **2** was further confirmed by DEPT, HSQC, COSY, NOESY, and HMBC experiments. Hence, it was determined to be 1β-hydroxy-8β-methoxy-eudesman-4(15), 7(11)-dien-8α,12-olide and was named as methylneolitacumone A.

Compound **3** was isolated as a white powder with [α]${}_{D}^{25}$ +225.2° (*c* 0.04, CHCl_3_). The molecular formula was obtained as C_15_H_18_O_3_ with ESIMS and HRESIMS analysis, which is two hydrogens less than neolitacumone B (**5**). Its IR and UV spectra exhibited absorption peaks attributable to an α,β-unsaturated lactone moiety [8,21]. According to ^1^H, ^13^C-NMR, and DEPT spectra, **3** was suggested to share a similar skeleton with neolitacumones A (**4**), B (**5**), and methylneolitacumone A (**2**) as an eudesmane-type sesquiterpenoid [8,21]. Comparison of compound **3** with **5** displayed similarities in both the physical data and the ^1^H and ^13^C-NMR spectra while the difference appeared at C-1 with a carbonyl group (IR: 1749 cm^−1^ and δ*c* 211.3) replacing a hydroxy group in **5**. Meanwhile, an 0.29 ppm downfield shift of the characteristic methyl signals at C-14 of eudesmanolide was due to a carbonyl inductive effect. The structure was also supported by HMBC correlations from H-9α (δ_H_ 1.44) and H-9β (δ_H_ 2.63) to C-1 and from H-14 (δ_H_ 1.17) to C-1. In addition, HMBC correlations from H-2α/H-2β and H-3α/H-3β to C-1 (δ 211.3); from H-13 to C-7 (δ 160.3), C-11 (δ 121.4), and C-12 (δ 174.5); from H-14 to C-1 (δ 211.3), C-5 (δ 48.2), C-9 (δ 40.0), and C-10 (δ 49.4); from H-15a and H-15b to C-3 (δ 34.4) and C-5 in **3** were employed for determining the planar structure of **3**. The β-orientation of H-8 and H-14 of **3** was due to NOESY cross-peaks between δ 4.80 (H-8)/1.17 (H-14) and 2.63 (H-9β), and δ 1.17 (H-14)/2.50 (H-6β) and 2.63 (H-9β). Accordingly, relative configurations of **3** were confirmed as 5α-H, 8β-H, and 10β-CH_3_. Meanwhile, optical rotation of **3** [α]${}_{D}^{25}$ +225.2° was similar to other eudesman-type compounds **2** and **4**–**6** as a positive endpoint, suggesting their relative configurations are similar [8]. As determined by the above observations, the structure of **3** was determined to be 1-oxoeudesman-4(15),7(11)-dien-8α,12-olide and was named neolitacumone E.

The known compounds, neolitacumones A–C (**4**–**6**) [8], β-sitosterol (**7**) [22], quercetin (**8**) [22], dihydroquercetin (**9**) [23], epicatechin (**10**) [22], oplopanone (**11**) [24], zeorin (**12**) [25], linderaggrine A (**13**) [26], clovane-2β,9α-diol (**14**) [27], and stigmast-5-ene-3β-yl formate (**15**) [28] were identified by comparison of their physical and spectroscopic data with literature values.

### 2.2. Anti-E. Coli β-Glucuronidase Activity of Compounds Isolated from N. acuminatissima

**Figure 3:**
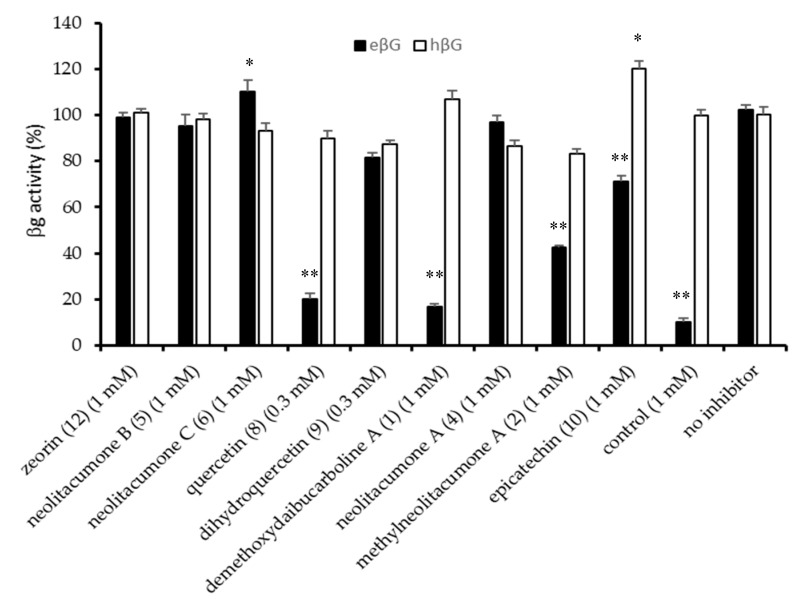
Anti-*E. coli* β-Glucuronidase (anti-eβG) activity of isolates of the root of *N. acuminatissima*. Each column represents the mean ± SD of three experiments. * *p* < 0.05, ** *p* < 0.01 indicates significant differences from the no inhibitor group.

Bioassay-guided isolation of NARD and NARE led to purification of 15 compounds. Among them, compounds **1**, **2**, **4**–**6**, **8**–**10**, and **12** were examined for their specific inhibition for eβG versus hβG by in vitro βG-based activity assays. The result showed that compounds **1** (1 mM) and **8** (0.3 mM) exhibited strong anti-eβG activity comparable to the positive control (1-((6,8-dimethyl-2-oxo-1,2-dihydroquinolin-3-yl)methyl)-3-(4-ethoxyphenyl)-1-(2-hydroxyethyl)thiourea, 1mM) [29], and led eβG activity below 20%, respectively (Figure 3). Compounds **2** and **10** had moderate or weak anti-eβG activity. Compounds **1** and **8** showed significant anti-eβG activity without affecting the enzyme activity of normal human intestinal (hβG > 90%); therefore, they do not influence normal function of human intestines. They may provide as a specific inhibitor to reduce eβG-induced intestinal injury and CID. Comparing the anti-eβG effect of flavone-quercetin (**8**), flavanone-dihydroquercetin (**9**), and flavan-epicatechin (**10**), these similar flavonoids with different saturations in the C-ring showed different behavior; compounds **9** and **10** had slight or weak anti-eβG effect, whereas **8** with a double bond between C-2/C-3 in conjugation with a 4-carbonyl group in ring C significantly decreased eβG activity. The result suggests that the flavone co-planar skeleton of **8** is better stabilized in the enzymes’ binding pocket than **9** and **10** [30]. Since the inhibitory effect of β-Glucuronidase of eudesmanolide-type sesquiterpenes has never been reported, in the present paper, the anti-eβG result of methylneolitacumone A (**2**), neolitacumone A (**4**), neolitacumone B (**5**), and neolitacumone C (**6**) with the same eudesmanolide skeleton were discussed. Compound **2** exhibited moderate anti-eβG activity, while compounds **4**–**6** did not affect the enzyme effect. Comparison of these compounds showed that compound **2** has a methoxy group instead of a hydroxy group at C-8 and showed a better inhibitory effect than **4**. Thus, it appears that presence of the lipophilic 8-methoxy group decreases eβG activity.

β-Glucuronidase is a lysosomal enzyme and has been found in animals, plants, and bacteria [31]. This enzyme is responsible for hydrolysis of β-glucuronide conjugates of endogenous and exogenous compounds in the body, such as benzo[*a*]pyrene glucuronides and natural plant glucuronides. It has been found that βG released from macrophages and neutrophils is necessary for bioactivation of glucuronide conjugates into the aglycone [30,32], and that the enzyme requires acidic conditions for its catalytic activity. For hβG, it exhibits maximal catalytic activity at pH 4–4.5, while eβG shows optimal activity at neutral pH [32,33,34]. Inhibition or dysfunction of hβG may disturb glycosaminoglycan degradation and cause mucopolysaccharidosis (MPS), affecting appearance, physical abilities, organ and system functioning, and mental development [34,35]. Thus, it is worthy to figure out candidates that can specifically inhibit eβG activity but not hβG.

CID is a common side effect experienced by cancer patients; it affects quality of life and may result in early death either directly from life-threatening sequelae or indirectly from adjustments in cancer treatment that result in suboptimal therapy [14,15,16]. Many studies indicate that inhibition of βG activity can reduce CPT-11-induced intestine mucosal damage and CID [19,20,33]. Although use of antibiotics against intestinal βG could relieve CPT-11-induced diarrhea, allergies and resistance effects must be consulted or monitored for choosing an antibiotic; meanwhile, antibiotics will kill all native gut floras, including probiotics within the digestive tract, which is not recommended for long-term use in chemotherapeutic patients [14,34]. Development of eβG-specific inhibitors can be used as a chemotherapy adjuvant to reduce CID [33].

To sum up these results, demethoxydaibucarboline A (**1**), methylneolitacumone A (**2**), and quercetin (**8**) isolated from *N. acuminatissima* may be candidate-specific eβG inhibitors to reduce CID and intestinal injury. The best inhibitor among these isolates is quercetin (**8**). As we know, quercetin is the major flavonoid in our diet. Diets rich in quercetin and other flavonoids may relate to decreasing incidence of cardiovascular, neoplastic, and neurodegenerative diseases [30,32,36]. Thus, a specific eβG inhibitor, such as quercetin, could be used as a nutrient supplement for chemoprevention and health promotion. Further experiments are needed to pinpoint their mechanisms of action.

## 3. Materials and Methods

### 3.1. General

Melting points were determined on an Electrothermal MEL-TEMP 3.0 apparatus (manufacturer, city, state abbrev. if USA, country) and were uncorrected. Optical rotations were recorded on a Jasco P2000 digital polarimeter. UV spectra were obtained in methanol on a Beckman Coulter TM-DU 800 UV–visible spectrophotometer. IR spectra were measured on a Perkin Elmer system 2000 FTIR spectrophotometer. 1D (^1^H, ^13^C, DEPT) and 2D (COSY, NOESY, HSQC, HMBC) NMR spectra using CDCl_3_, acetone-*d_6_*, or methanol-*d_4_* as solvents were recorded on Varian Gemini-2000 200 MHz, Varian Unity Plus 400 MHz, Mercury Plus 400 MHz, and Varian VNMRS 600 MHz FT-NMR spectrometers. Deuterated solvents were purchased from Sigma-Aldrich (St. Louis, MO, USA). Low-resolution ESIMS was obtained with Waters ZQ 4000 and JEOL-JMS-HX 100 mass spectrometers. High-resolution ESIMS was recorded on JEOL JMS-SX102A GC/LC/MS and Finnigan MAT-95XL mass spectrometers. Silica gel (70–230 Mesh; Merck, Darmstadt, Germany) and spherical C_18_ 100 Å reversed-phase silica gel (RP-18; particle size 40–63 μm; Silicycle, Quebec, Canada) were used for column chromatography, and silica gel 60 F254 (Merck) and RP-18 F254S (Merck) were used for TLC and preparative TLC. Further purification was performed by HPLC (Chrom Tech P230 HPLC pump, Phenomenex Luna 5u Silica 250 × 10.00 mm, 5 micron, refractive index detector IOTA2). The reagents in the β-Glucuronidase activity assay, e.g., bovine serum albumin, *p*-nitrophenyl-β-d-glucopyranoside, and 1-((6,8-dimethyl-2-oxo-1,2-dihydroquinolin-3-yl)methyl)-3-(4-ethoxyphenyl)-1-(2-hydroxyethyl)thiourea were all purchased from Sigma-Aldrich (St. Louis, MO, USA).

### 3.2. Plant Material

Roots of *N. acuminatissima* were collected in July 2012 from Yilan County, Taiwan and positively identified by one of the authors, Prof. I. S. Chen. A voucher specimen (2012-07-NA) was deposited at the Herbarium of the Department of Fragrance and Cosmetic Science, Kaohsiung Medical University, Kaohsiung, Taiwan.

### 3.3. Extraction and Isolation

Air-dried roots of *N. acuminatissima* (7.4 kg) were extracted three times with MeOH (20 L) at room temperature. The extracts were filtered and concentrated under reduced pressure, and the residue (NARM, 440 g) was successively partitioned with dichloromethane, ethyl acetate, *n*-butanol, and water to obtain four different soluble fractions, named NARD (87.7 g), NARE (30.3 g), NARB (198.0 g), and NARW (68.8 g), respectively. All were assessed by anti-eβG activity. Active NARD and NARE layers were further purified using silica gel column chromatography (CC).

NARD (87.7 g) was subjected to silica gel CC with a gradient of *n*-hexane/acetone/MeOH to obtain fractions 1–14. Fractions 9, 10, 12, and 14 showed anti-eβG activity. Fraction 3 (26.5 g) was purified by silica gel CC, eluting with *n*-hexane/CH_2_Cl_2_/acetone (2:2:1), MeOH/H_2_O (10:0.1), and *n*-hexane/acetone (3:1) to individually yield **12** (120.5 mg), **7** (165.0 mg) and **15** (2.0 mg). Fraction 4 (3.2 g) was subjected to CC, eluting with *n*-hexane/EtOAc and *n*-hexane/acetone (6:1) then recrystallized to yield **6** (74.0 mg). In addition, **11** (1.2 mg) was also obtained with eluting *n*-hexane/EtOAc and cyclohexane/CH_2_Cl_2_/acetone (13:1:1). Fraction 5 (2.3 g) was purified by silica gel CC eluting with CH_2_Cl_2_/acetone (15:1) and preparative HPLC eluting with *n*-hexane/acetone (3:1) to obtain **3** (1.1 mg). Fraction 7 (5.6 g) was subjected to CC eluting with *n*-hexane/CH_2_Cl_2_/acetone (1:3:2) and MeOH/H_2_O (1:1) to afford **4** (206.0 mg). In addition, **14** (2.0 mg) was also obtained with eluting *n*-hexane/CH_2_Cl_2_/acetone (1:3:1), *n*-hexane/CH_2_Cl_2_/acetone (2:2:1), MeOH/H_2_O (1:1), and *n*-hexane/acetone (3:1). Active fraction 9 (3.2 g) was chromatographed on CC, eluting with MeOH/H_2_O (1:1), *n*-hexane/acetone (6:1), and Sephadex LH-20 to afford **1** (12.1 mg). Active fraction 10 (6.4 g) was subjected to CC and recrystallized to obtain **5** (25.0 mg).

NARE (30.3 g) was subjected to CC with a gradient of MeOH/H_2_O (1:2–1:1–100% MeOH) to obtain fractions 1–10. Fractions 5–7 showed anti-eβG activity. Fraction 3 (0.8 g) was purified by CC, eluting with CH_2_Cl_2_/acetone (1:1) and MeOH/H_2_O (1:1) to yield **9** (53.6 mg). In addition, **10** (40.3 mg) and **1** (4.4 mg) were also obtained by silica gel CC individually eluting with CH_2_Cl_2_/acetone (2:1) and MeOH/H_2_O (1:1). Active fraction 5 (2.3 g) was purified by CC, eluting with *n*-hexane/acetone (1:2) and CH_2_Cl_2_/acetone (3:1) to obtain **13** (1.5 mg). Sequentially, **4** (14.0 mg) was afforded by silica gel CC eluting with *n*-hexane/EtOAc (1:1) and CH_2_Cl_2_/EtOAc (1:1). Active fraction 6 (2.0 g) was subjected to Sephadex LH-20 and silica gel CC, eluting with *n*-hexane/EtOAc (1:3), *n*-hexane/acetone (3:1), and MeOH/H_2_O (1:1) to obtain **2** (6.8 mg). Fraction 9 (0.2 g) was purified by Sephadex LH-20 and CC, eluting with *n*-hexane/acetone (2:1) to yield **8** (18.7 mg).

#### 3.3.1. Demethoxydaibucarboline A (**1**)

Yellowish powder; UV (MeOH) λ_max_ (log *ε*) 228 (4.22), 241 (4.03), 297 (3.96), 356 (3.65), and 385 (sh, 3.42) nm; IR (KBr) *ν*_max_ 3277 (OH), 1610, 1583, and 1512 (aromatic ring) cm^−1^; ^1^H (400 MHz) and ^13^C (100 MHz) NMR spectral data, see Table 1; HRESIMS [M + H]^+^ *m*/*z* 291.1127 (calculated for C_18_H_15_N_2_O_2_, 291.1128).

#### 3.3.2. Methylneolitacumone A (**2**)

Colorless needles (acetone/water); m.p.: 112–114 °C; [α]${}_{D}^{25}$ + 148.6° (*c* 0.27, CHCl_3_); UV (MeOH) λ_max_ (log *ε*) 219 (3.94) nm; IR (KBr) *ν*_max_ 3419 (OH), 1733 (C=O), 1643 (C=CH_2_) cm^−1^; ^1^H (400 MHz) and ^13^C (100 MHz) NMR spectral data, see Table 1; HRESIMS [M + Na]^+^ *m*/*z* 301.1409 (calculated for C_16_H_22_O_4_Na, 301.1410).

#### 3.3.3. Neolitacumone E (**3**)

White powder; [α]${}_{D}^{25}$ +225.2° (*c* 0.04, CHCl_3_); UV (MeOH) λ_max_ (log *ε*) 218 (3.96) nm; IR (KBr) *ν*_max_ 1749, 1708 (C=O) cm^−1^; ^1^H (500 MHz) and ^13^C (125 MHz) NMR spectral data, see Table 1; HRESIMS [M + Na]^+^ *m*/*z* 269.1146 (calculated for C_15_H_18_O_3_Na, 269.1148).

### 3.4. In Vitro β-Glucuronidase Activity Assay

The effect of isolates of *N. acuminatissima* to inhibit β-Glucuronidase activity was determined using purified eβG and hβG enzymes obtained as described [34,35]. The enzymes were prepared in a reaction buffer (10% DMSO + 0.05% bovine serum albumin in PBS) at pH 7.5 and 4.5 for eβG and hβG, respectively. The test samples or vehicle control (10 μL) were mixed with 40 μL βG enzyme (3.75 ng/well) into a 96-well plate at 37 °C for 30 min and then 50 μL *p*-nitrophenyl-β-d-glucopyranoside (*p*NPG) (10 mM) was added and incubated at 37 °C for 1 h. Finally, 5 μL 2N NaOH was added to stop the reaction. Optical densities were determined at 405 nm using a microplate spectrophotometer. Results are displayed as percent of βG activity compared with the untreated control. 1-((6,8-dimethyl-2-oxo-1,2-dihydroquinolin-3-yl)methyl)-3-(4-ethoxyphenyl)-1-(2-hydroxy- ethyl)thiourea was used as a positive control. Data are shown as the mean ± standard deviation (SD) of three measurements. Groups were compared using the Student’s t-test (SPSS 18 Inc., UAS). Differences between groups were determined to be significant when * *p* < 0.05 and ** *p* < 0.01.

## 4. Conclusions

In this study, one new carboline alkaloid, demethoxydaibucarboline A (**1**), two new eudesmanolide-type sesquiterpenes, methylneolitacumone A (**2**), neolitacumone E (**3**), and twelve known compounds (**4**–**15**) were isolated from the root of *N. acuminatissima.* These compounds were investigated by using an anti-eβG assay. The results indicated that two isolated compounds, demethoxydaibucarboline A (**1**) and quercetin (**8**) showed significant anti-eβG activity with an inhibition ratio of approximately 80%, respectively. Methylneolitacumone A (**2**) exhibited a moderate inhibitory effect and eβG activity was less than 45%. Compounds **1** at 1 mM and **8** at a lower concentration of 0.3 mM exhibited specific inhibition of eβG activity but not hβG, suggesting that active secondary metabolites of *N. acuminatissima* are potential β-Glucuronidase inhibitors. These secondary metabolites will be potential β-Glucuronidase inhibitors that protect intestines from injury and thus relieve chemotherapy-induced diarrhea (CID). Although the detailed mechanism of action of these compounds remains to be determined, the results confirmed that *N. acuminatissima* is a valuable source from which natural product-based supplements and medicinal products can be derived.

## Figures and Tables

**Table 1:** ^1^H- and ^13^C-NMR spectroscopic data of compounds **1**–**3**.

Position	1 (Acetone-*d*_6_)		Position	2 (CDCl_3_)		3 (CDCl_3_)	
	δ_H_ (*J* in Hz)	δ_C_		δ_H_ (*J* in Hz)	δ_C_	δ_H_ (*J* in Hz)	δ_C_
1		146.6	1	3.36, dd (11.2, 4.0)	78.8		211.3
3	8.24, d (5.2)	139.0	2α	1.80, ddd (13.6, 4.8, 2.0)	30.8	2.73, td (16.1, 7.3)	37.3
4	7.82, d (5.2)	114.3	2β	1.57, ddd (13.6, 4.8, 2.0)		2.47, m	
5	7.56, d (2.4)	107.2	3α	2.05, td (13.8, 4.8)	33.6	2.67, td (14.6, 5.0)	34.4
6		152.8	3β	2.31, td (13.8, 4.8)		2.47, m	
7	7.10, dd (8.8, 2.4)	119.4	4		146.6		144.6
8	7.40, d (8.8)	113.9	5	1.88, m	49.6	2.22, dt (13.7, 1.6)	48.2
1a		131.3	6α	2.38, dd (13.0, 3.2)	24.2	2.86, dd (13.7, 3.6)	25.0
4a		129.7	6β	2.47, t (13.0)		2.50, td (13.7, 1.2)	
5a		123.9	7		158.8		160.3
8a		136.6	8		106.2	4.80, dd (11.8, 6.3)	78.2
1′		131.6	9α	1.38, d (13.6)	46.4	1.44, dd (13.2, 11.8)	40.0
2′, 6′	7.20, d (8.8)	113.3	9β	2.78, d (13.6)		2.63, td (13.2, 6.3)	
3′, 5′	6.70, d (8.8)	116.6	10		41.1		49.4
4′		157.3	11		124.3		121.4
7′	4.37, s	40.8	12		171.7		174.5
NH	10.32, br s		13	1.86, d (1.6)	8.3	1.83, t (1.6)	8.6
			14	0.94, s	10.3	1.17, s	17.0
			15a	4.64, d (1.6)	108.1	4.92, s	110.8
			15b	4.90, d (1.6)		5.16, s	
			OCH_3_	3.16, s	50.4		

^1^H- (400 MHz) and ^13^C-NMR (100 MHz) data for compounds **1** and **2**; ^1^H- (500 MHz) and ^13^C-NMR (125 MHz) data for compound **3**.

## Supplementary Materials

The following are available online. Figure S1: Preliminary anti-eβG screening assay of partial Lauraceae plants. Figures S2–S28: The 1D- and 2D-NMR spectra of compounds **1**–**3**.
