# Supplementary material for: Chemical Constituent of β-Glucuronidase Inhibitors from the Root of Neolitsea acuminatissima
Source: Molecules. 2020 Nov 6;25(21):5170. doi: 10.3390/molecules25215170 (PMC7664238; doi:10.3390/molecules25215170)
Supplement: Supplementary file 1 [file molecules-25-05170-s001.pdf]

Chemical constituent of β-glucuronidase inhibitors from the root of *Neolitsea acuminatissima*

Chu-Hung Lin [1,†], Hsiao-Jung Chou [2,†], Chih-Chi Chang [2], Ih-Sheng Chen[3], Hsun-Shuo Chang[3,5], Tian-Lu Cheng [4,5], Yueh-Hiung Kuo [6,7,8,*], and Horng-Huey Ko [2,5,*]

[1] Herbal Medicinal Product Technology Division, Biomedical Technology and Device Research Laboratories, Industrial Technology Research Institute, Hsinchu 30011, Taiwan; chuhung.lin@gmail.com

[2] Department of Fragrance and Cosmetic Science, College of Pharmacy, Kaohsiung Medical University, Kaohsiung 80708, Taiwan; cc032324@gmail.com (H.-J.C.); newheart1920@hotmail.com (C.-C.Chang)

[3] School of Pharmacy, College of Pharmacy, Kaohsiung Medical University, Kaohsiung 807, Taiwan; hschang@kmu.edu.tw (H.-S.C.); m635013@kmu.edu.tw (I.-S.C.)

[4] Department of Biomedical Science and Environmental Biology, Kaohsiung Medical University, Kaohsiung 80708, Taiwan

[5] Drug Development and Value Creation Center, Kaohsiung Medical University, Kaohsiung 807, Taiwan

[6] Department of Chinese Pharmaceutical Sciences and Chinese Medicine Resources, China Medical University, Taichung 40402, Taiwan

[7] Chinese Medicine Research Center, China Medical University, Taichung 40402, Taiwan

[8] Department of Biotechnology, Asia University, Taichung 41354, Taiwan

* Correspondence: hhko@kmu.edu.tw (H.-H.K.); kuoyh@mail.cmu.edu.tw (Y.-H.K.); Tel.: +886-7-3121101 ext. 2643; fax: +886-7-3210683 (H.-H.K.)

† These authors contributed equally to this work.

The List of Supplementary Material

Figure S1	Preliminary anti-eβG screening assay of partial Lauraceae plants…..	4
Figure S2	FT-IR spectrum of **1**………………………………………………….	5
Figure S3	^{1}H NMR spectrum of **1** (400 MHz, acetone-d_6)………………………..	5
Figure S4	^{13}C NMR spectrum of **1** (100 MHz, acetone-d_6)………………………..	6
Figure S5	DEPT spectrum of **1**……………………………………………………	6
Figure S6	HSQC spectrum of **1**………………………...………………………....	7
Figure S7	HMBC spectrum of **1**……………………..	7
Figure S8	COSY spectrum of **1**………………………..………………………….....	8
Figure S9	NOESY spectrum of **1**………………………...………………………..	8
Figure S10	HRESIMS spectrum of **1**………………………...…………………….	9
Figure S11	FT-IR spectrum of **2**………………………………………………….	9
Figure S12	^{1}H NMR spectrum of **2** (400 MHz, $CDCl_3$)…………………………....	10
Figure S13	^{13}C NMR spectrum of **2** (100 MHz, $CDCl_3$)……………………….........	10
Figure S14	DEPT spectrum of **2**……………………………………………………	11
Figure S15	HSQC spectrum of **2**………………………...………………………....	11
Figure S16	HMBC spectrum of **2**……………………..	12
Figure S17	COSY spectrum of **2**………………………..…………………………..	12
Figure S18	NOESY spectrum of **2**………………………...………………………..	13
Figure S19	HRESIMS spectrum of **2**………………………...…………………….	13
Figure S20	FT-IR spectrum of **3**………………………………………………….	14
Figure S21	^{1}H NMR spectrum of **3** (500 MHz, $CDCl_3$)…………………………....	14
Figure S22	^{13}C NMR spectrum of **3** (125 MHz, $CDCl_3$)……………………….........	15
Figure S23	DEPT spectrum of **3**……………………………………………………	15
Figure S24	HSQC spectrum of **3**………………………...………………………....	16

Figure S25 HMBC spectrum of **3**…………………….. 16

Figure S26 COSY spectrum of **3**………………………..………………………..... 17

Figure S27 NOESY spectrum of **3**………………………...……………………….. 17

Figure S28 HRESIMS spectrum of **3**………………………...…………………….. 18

Preliminary anti-eβG screening assay of partial Lauraceae plants

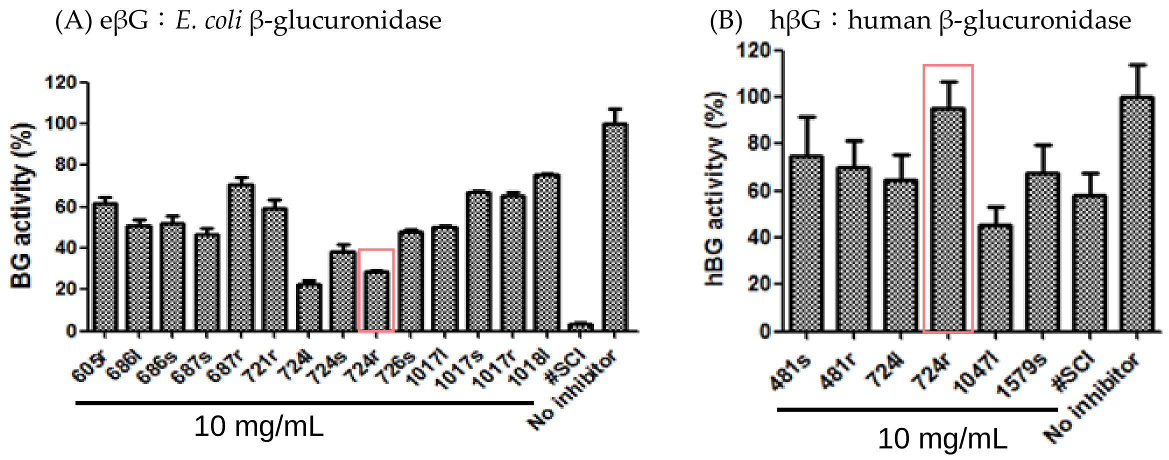

Figure S1 (A) Anti-eβG activity of different samples and (B) anti-hβG activity in human intestines. 724r is the methanolic extract of the root of *N. acuminatissima*. #SCI: 1-((6,8-dimethyl-2-oxo-1,2-dihydroquinolin-3-yl) methyl)-3-(4-ethoxyphenyl)-1-(2-hydroxyethyl) thiourea was used as positive control.

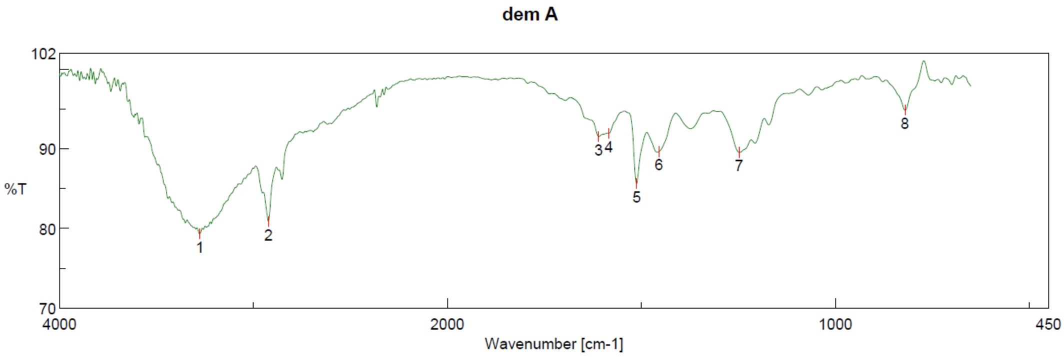

[Result of Peak Picking]

No.	Position	Intensity	No.	Position	Intensity	No.	Position	Intensity
1	3277.43	79.2999	2	2923.56	80.8587	3	1610.27	91.4946
4	1583.27	91.9341	5	1511.92	85.5947	6	1455.03	89.6202
7	1247.72	89.5222	8	820.563	94.8331			

Figure S2 FTIR spectrum of **1**

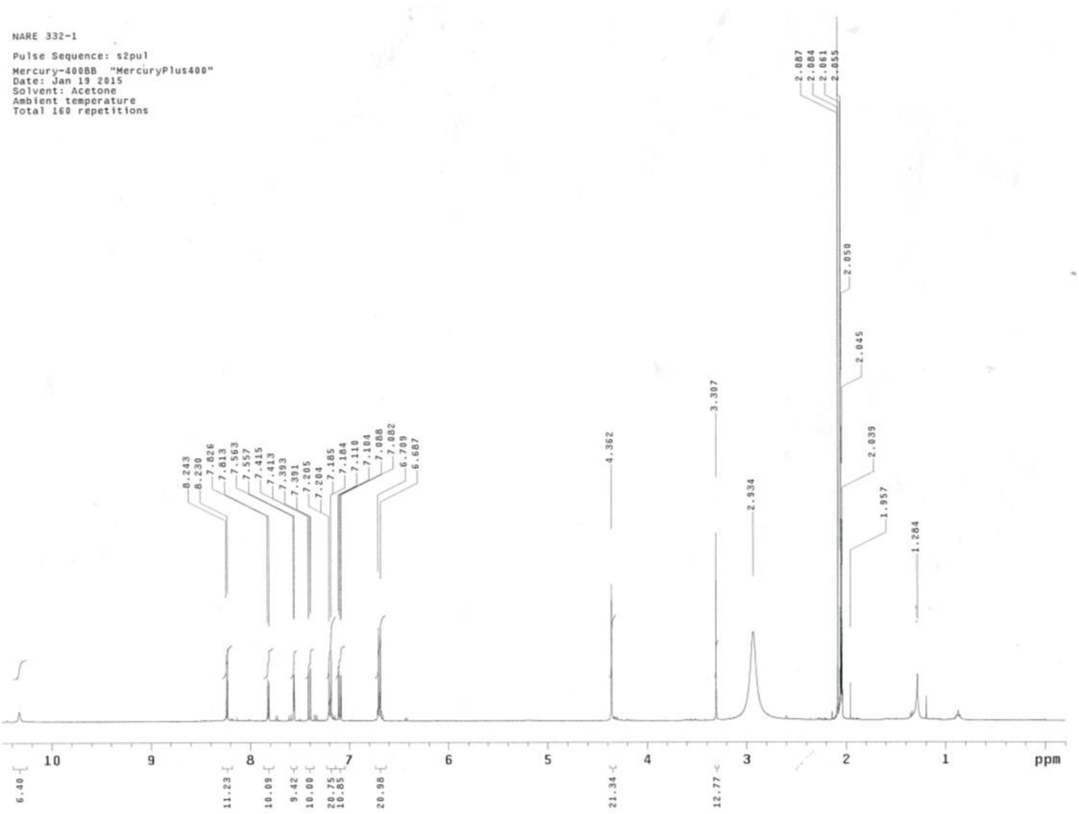

Figure S3 ^{1}H NMR spectrum of **1** (400 MHz, acetone-d_6)

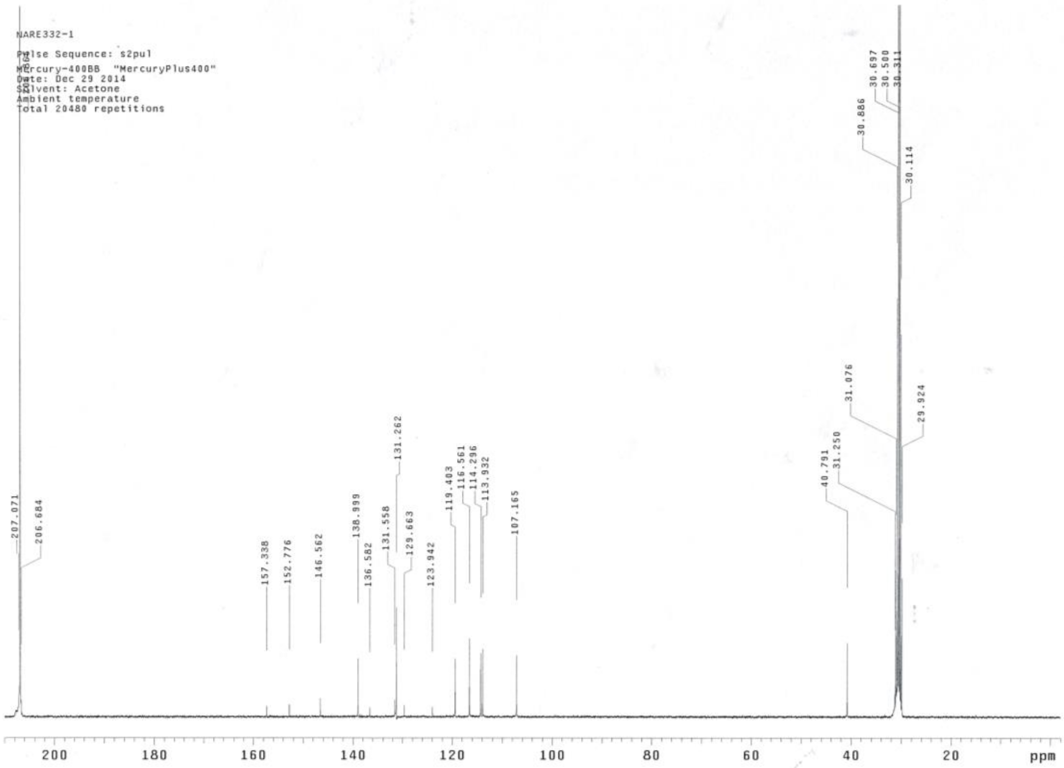

Figure S4 ^{13}C NMR spectrum of **1** (100 MHz, acetone-d_6)

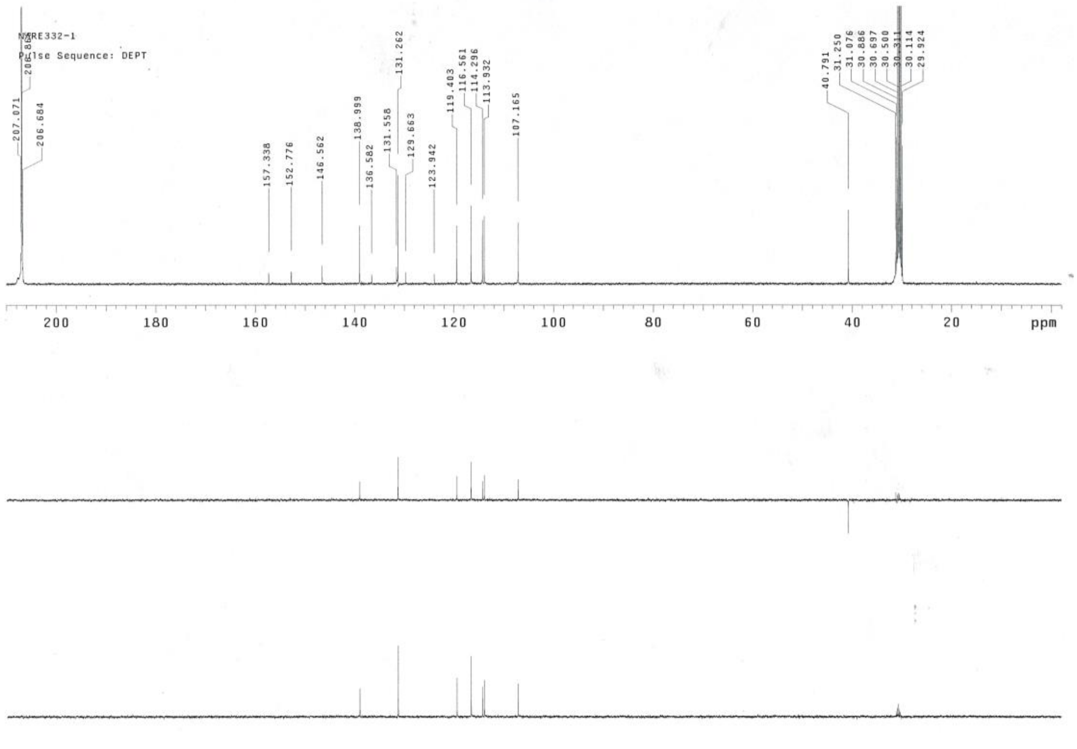

Figure S5 DEPT spectrum of **1**

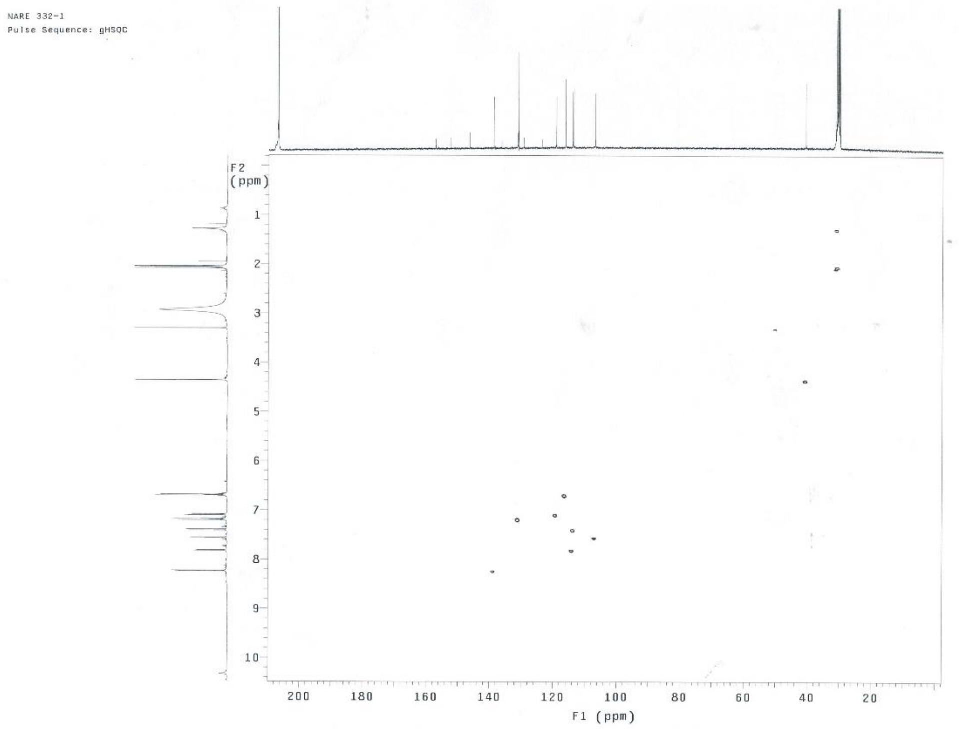

Figure S6 HSQC spectrum of **1**

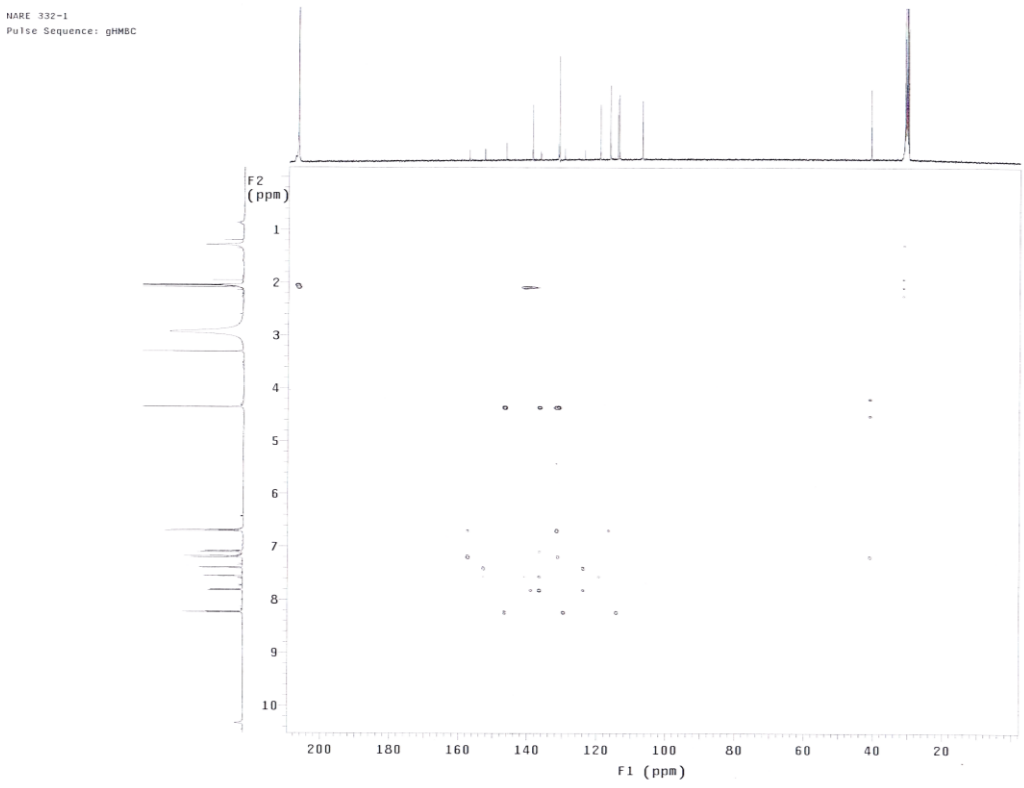

Figure S7 HMBC spectrum of **1**

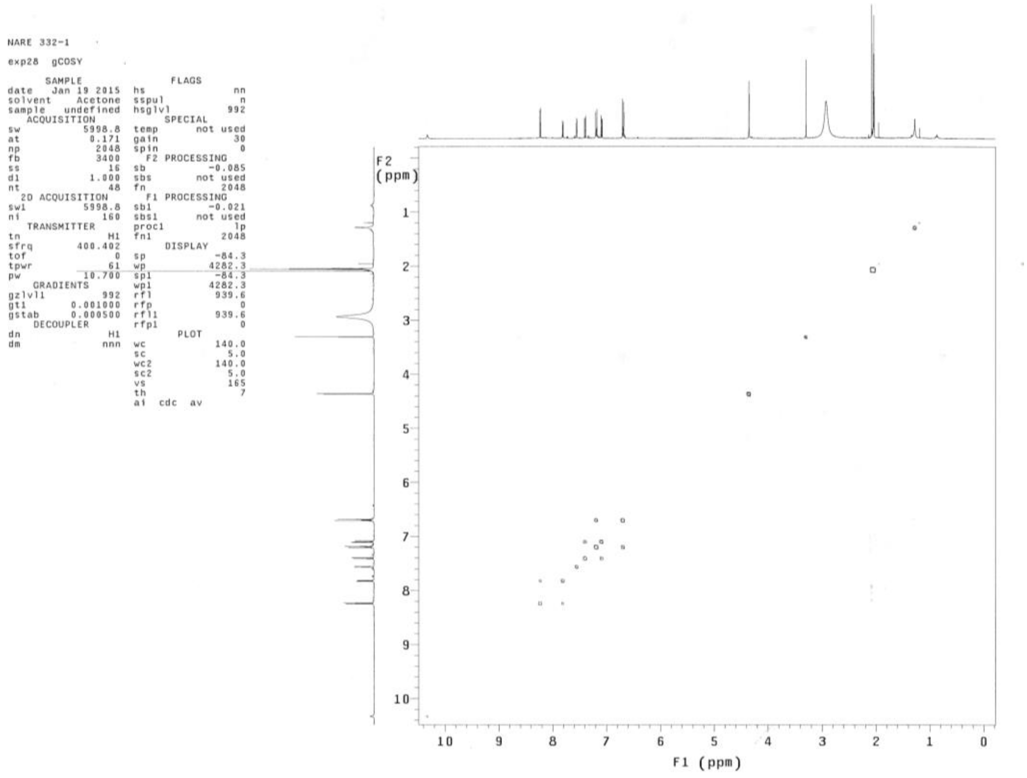

Figure S8 COSY spectrum of **1**

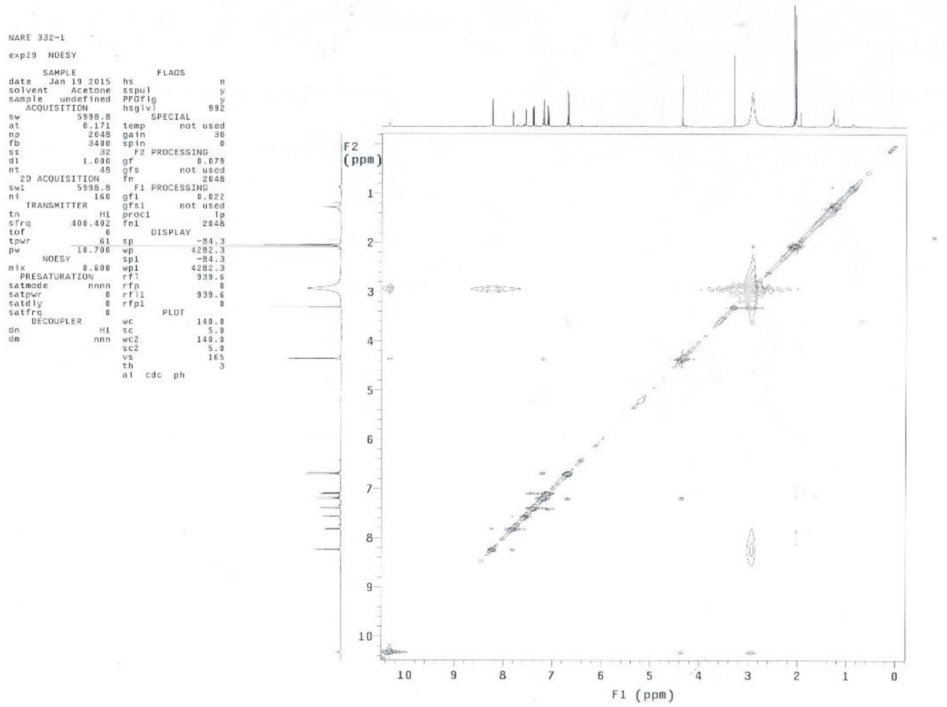

Figure S9 NOESY spectrum of **1**

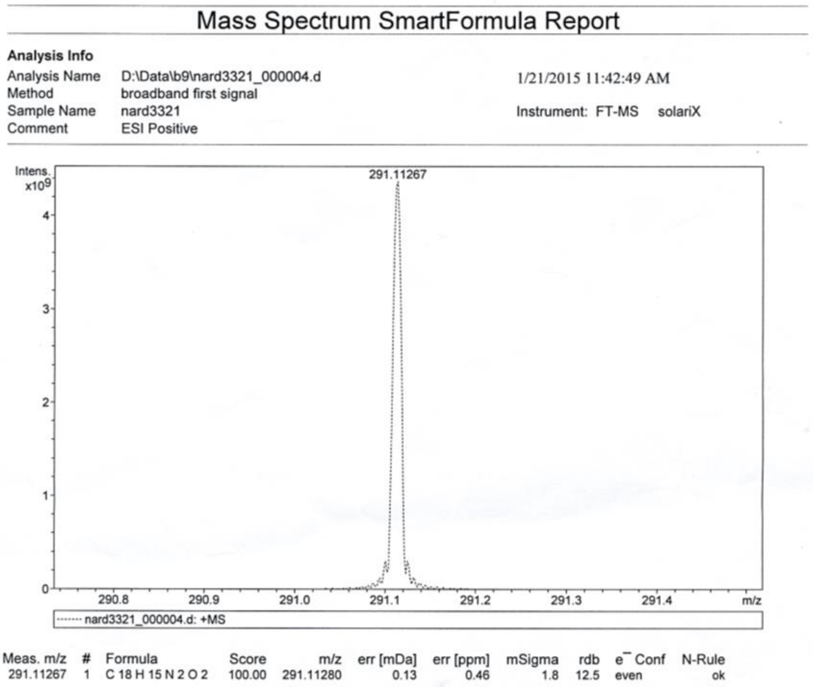

Figure S10 HRESIMS spectrum of **1**

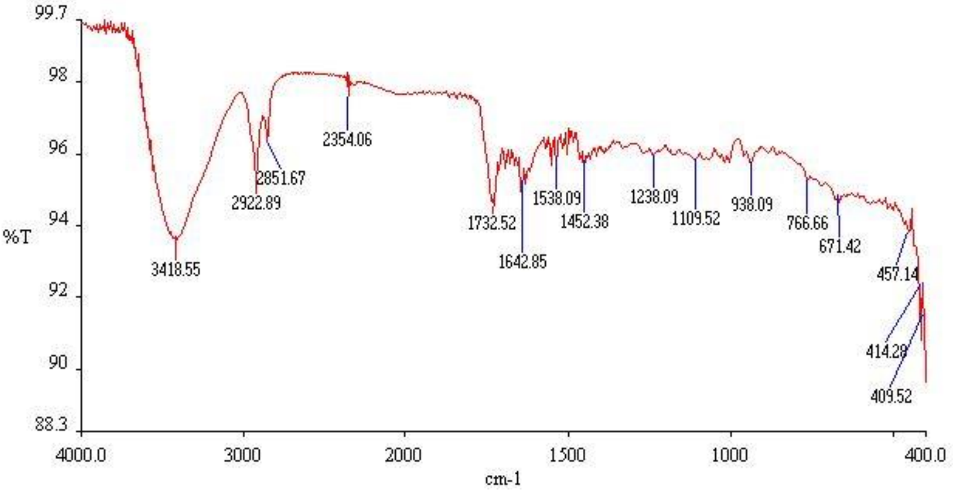

Figure S11 FT-IR spectrum of **2**

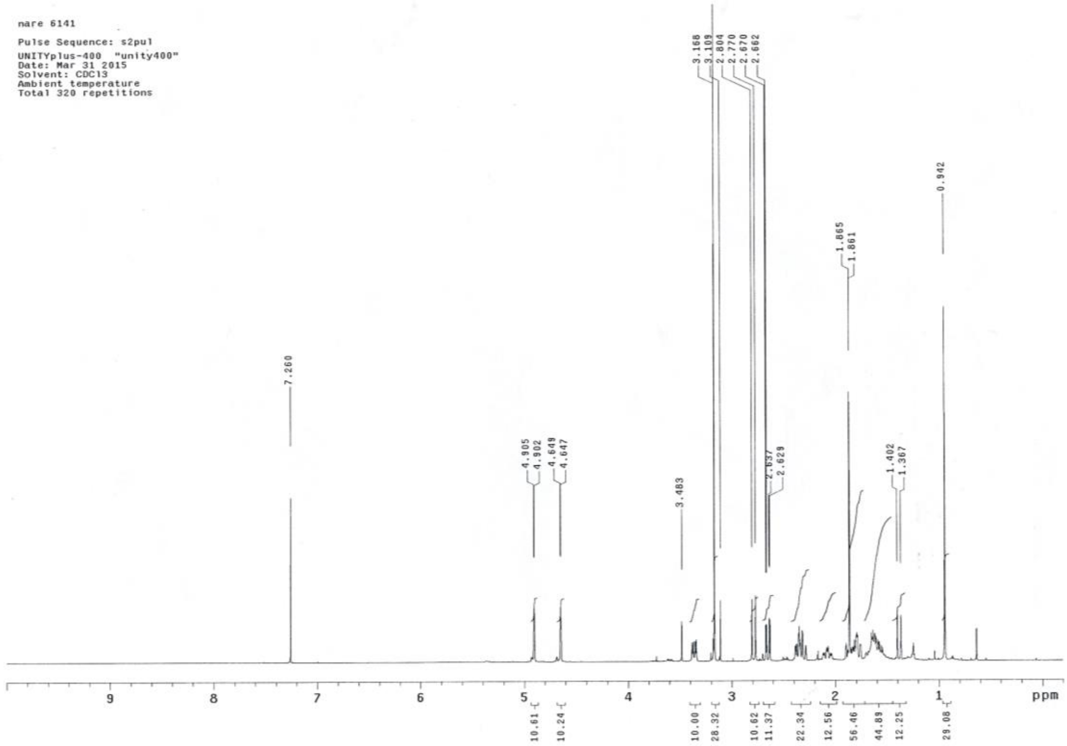

Figure S12 ^{1}H NMR spectrum of **2** (400 MHz, $CDCl_3$)

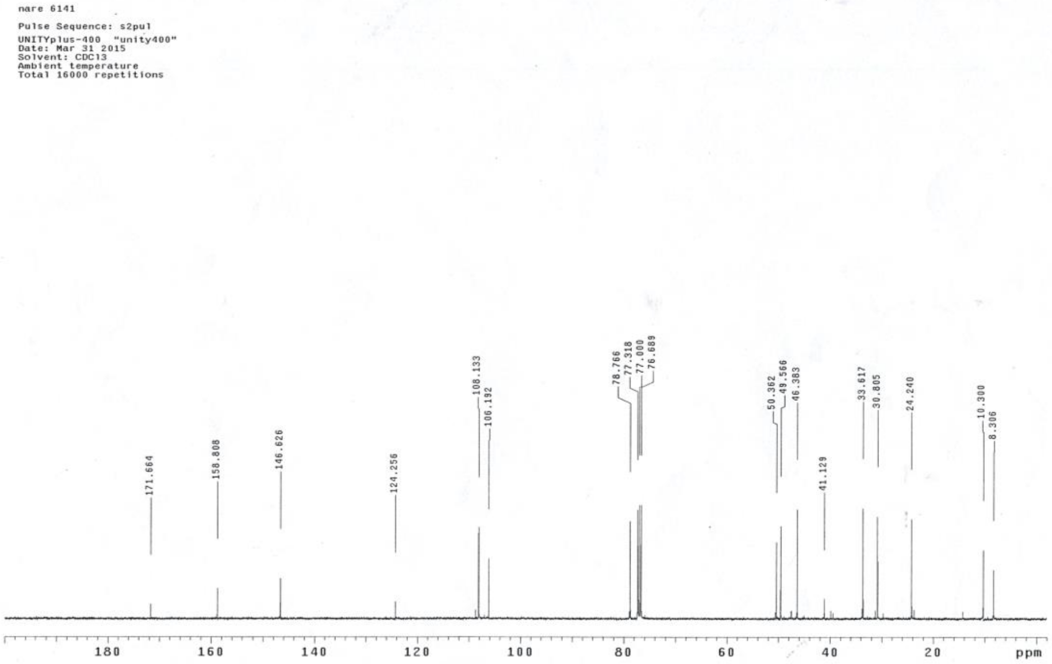

Figure S13 ^{13}C NMR spectrum of **2** (100 MHz, $CDCl_3$)

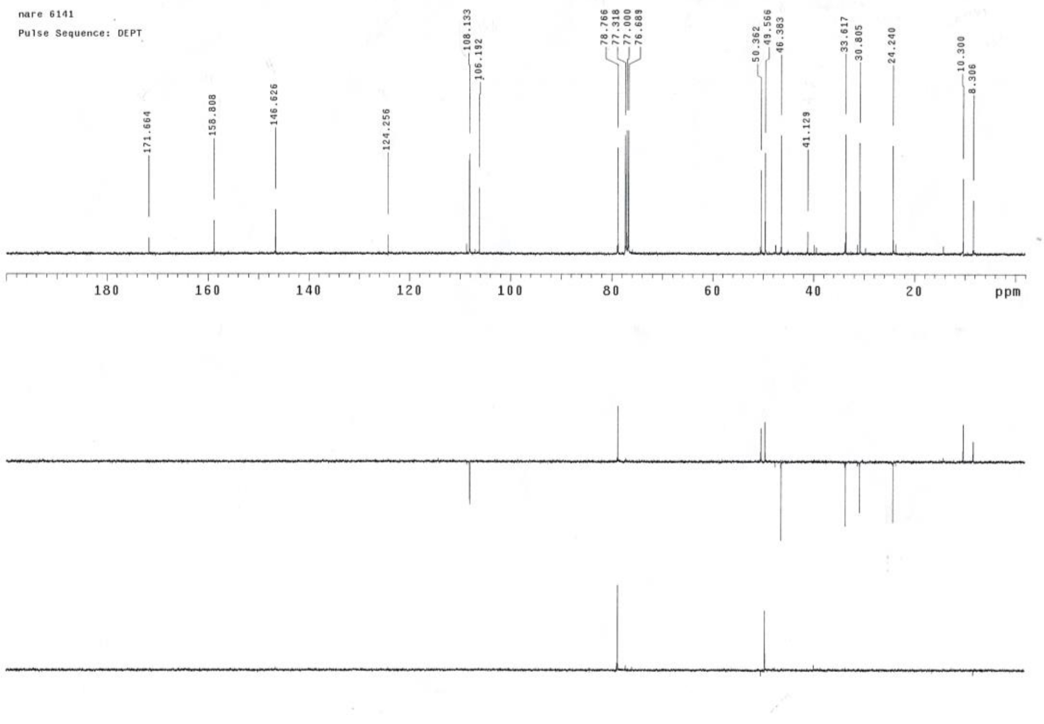

Figure S14 DEPT spectrum of **2**

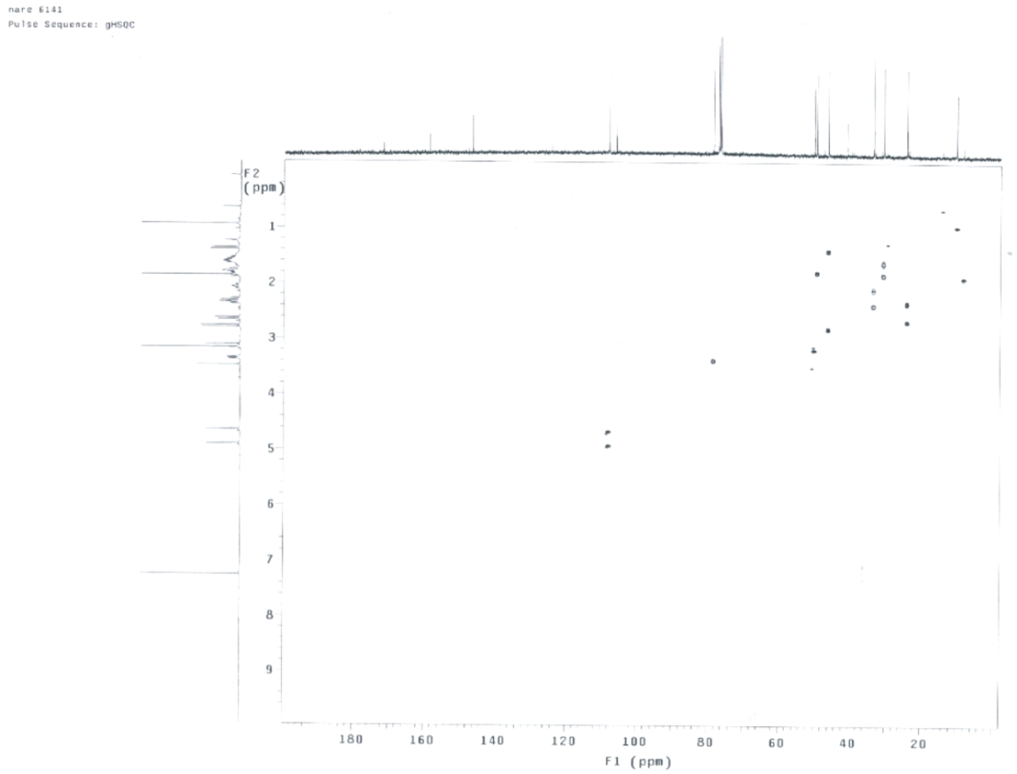

Figure S15 HSQC spectrum of **2**

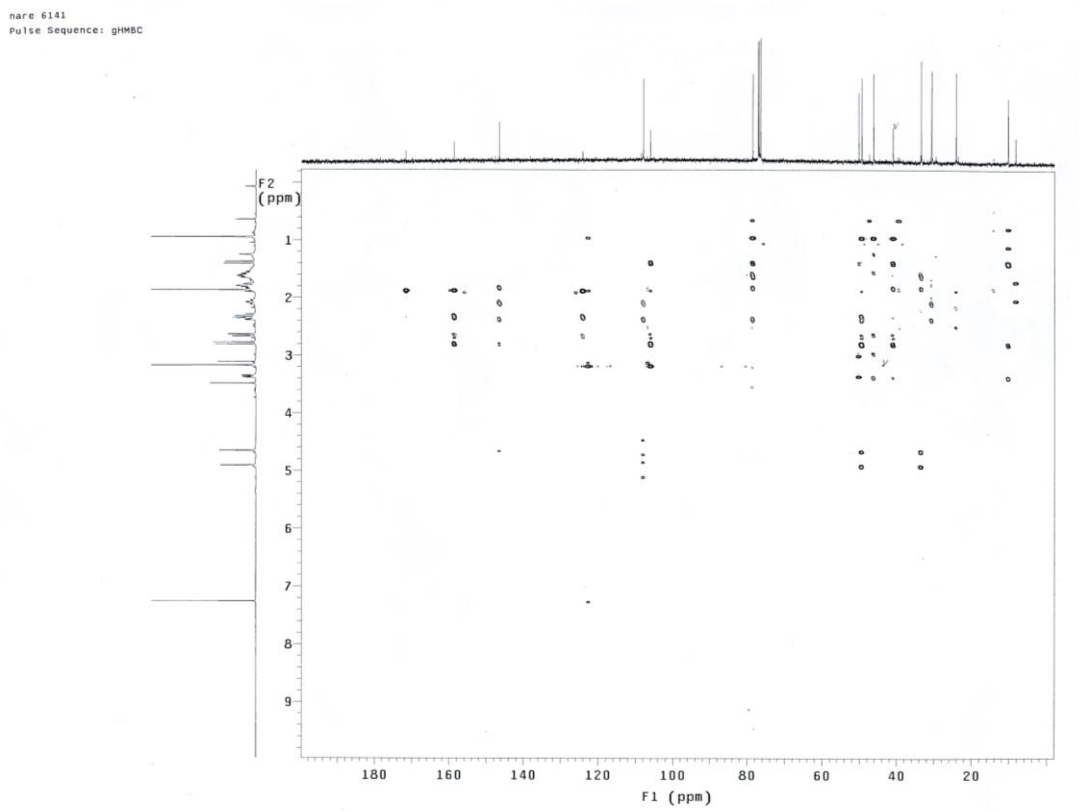

Figure S16 HMBC spectrum of **2**

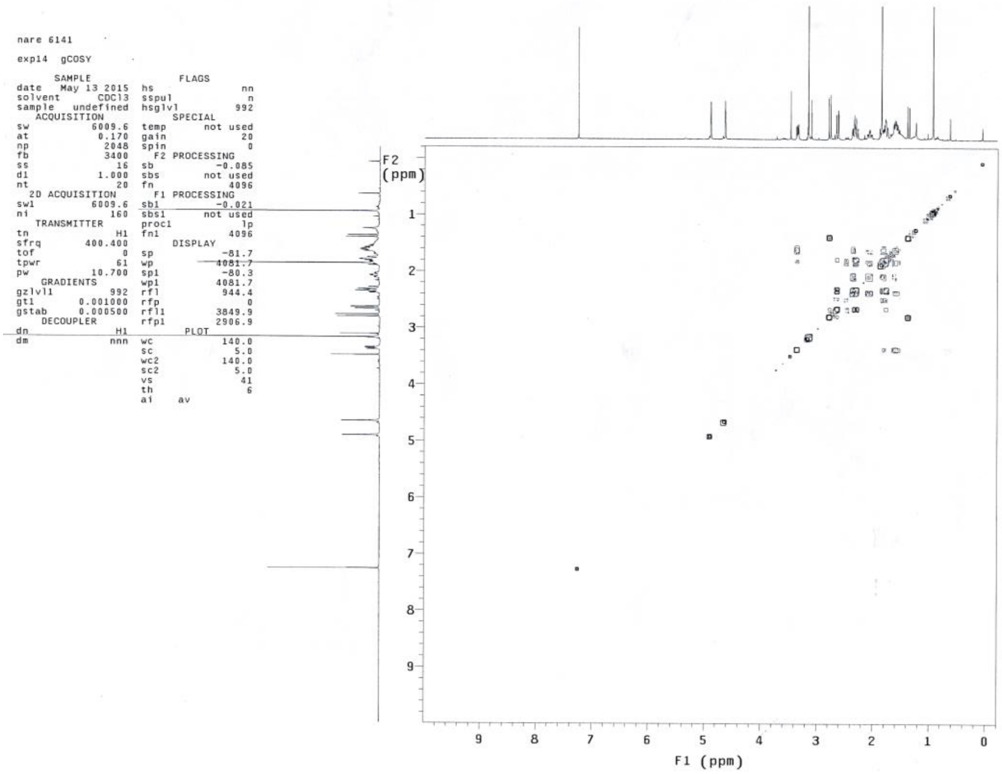

Figure S17 COSY spectrum of **2**

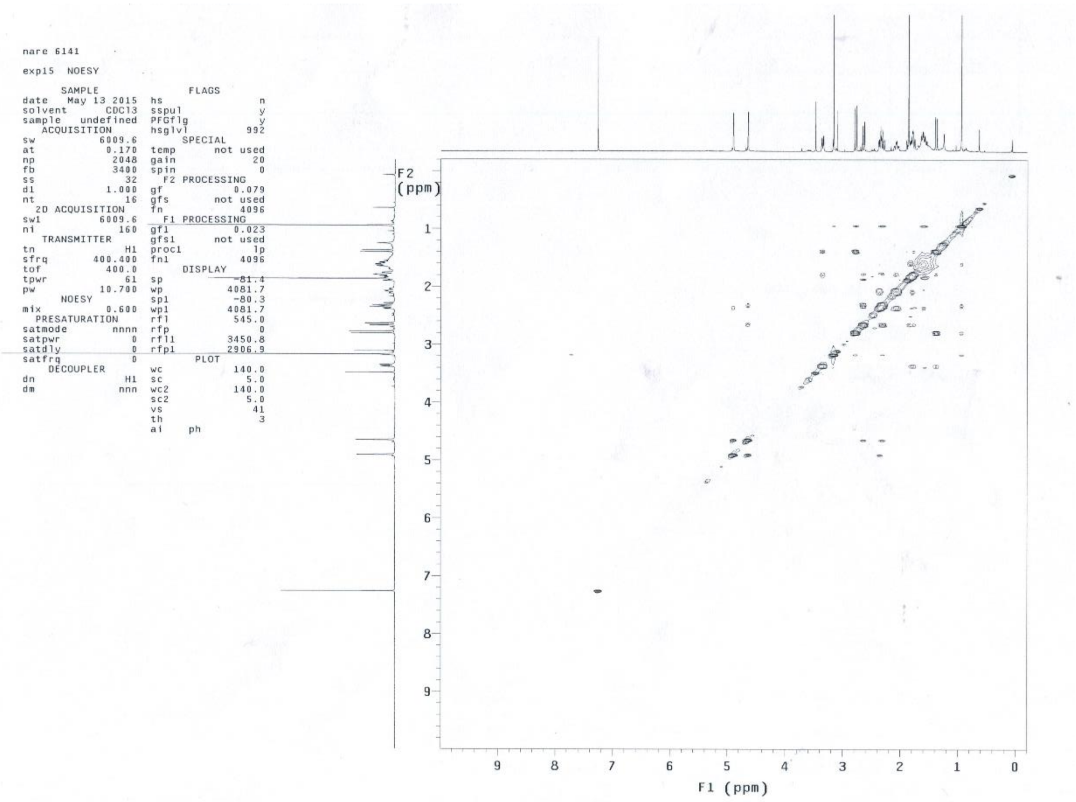

Figure S18 NOESY spectrum of **2**

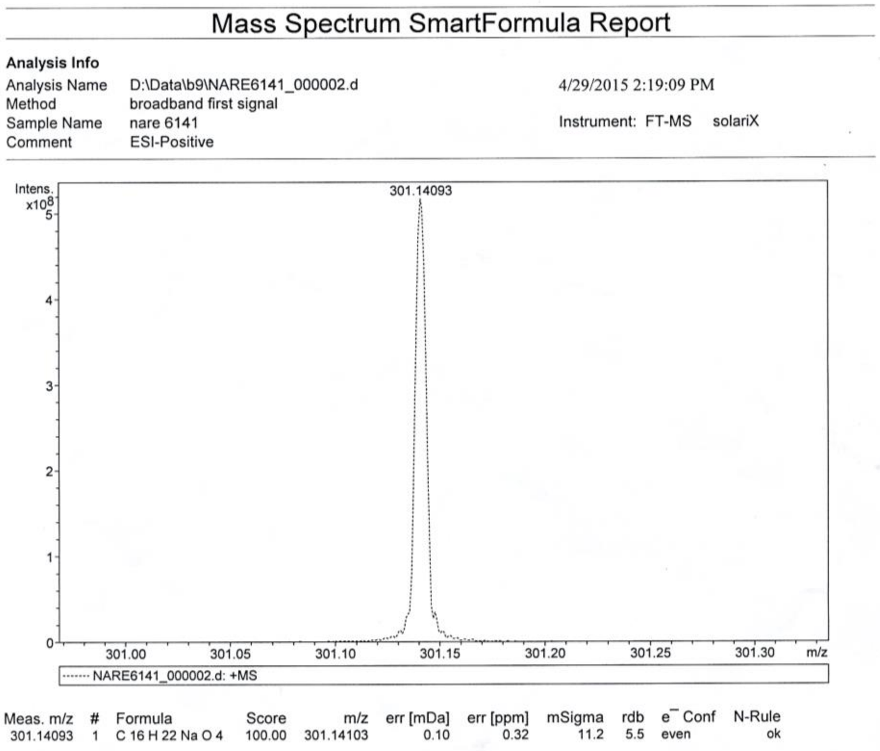

Figure S19 HRESIMS spectrum of **2**

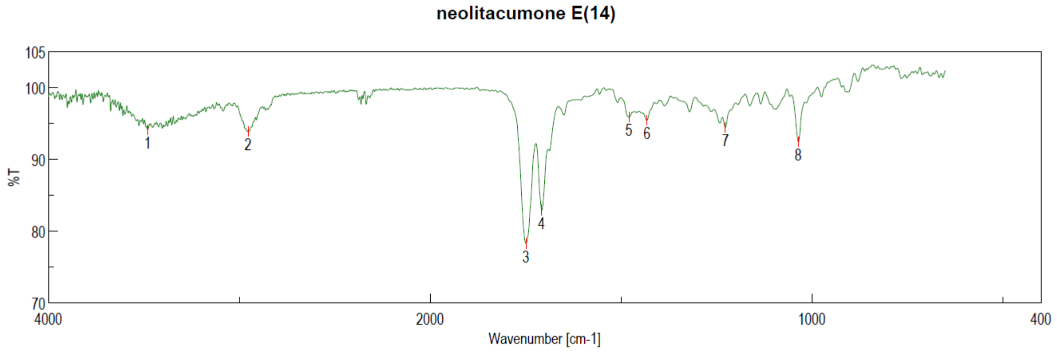

[Result of Peak Picking]

No.	Position	Intensity	No.	Position	Intensity	No.	Position	Intensity
1	3478.95	94.0693	2	2954.41	93.8672	3	1749.12	78.2845
4	1707.66	82.9194	5	1479.13	95.9004	6	1431.89	95.4234
7	1226.5	94.4163	8	1035.59	92.4426			

Figure S20 FT-IR spectrum of **3**

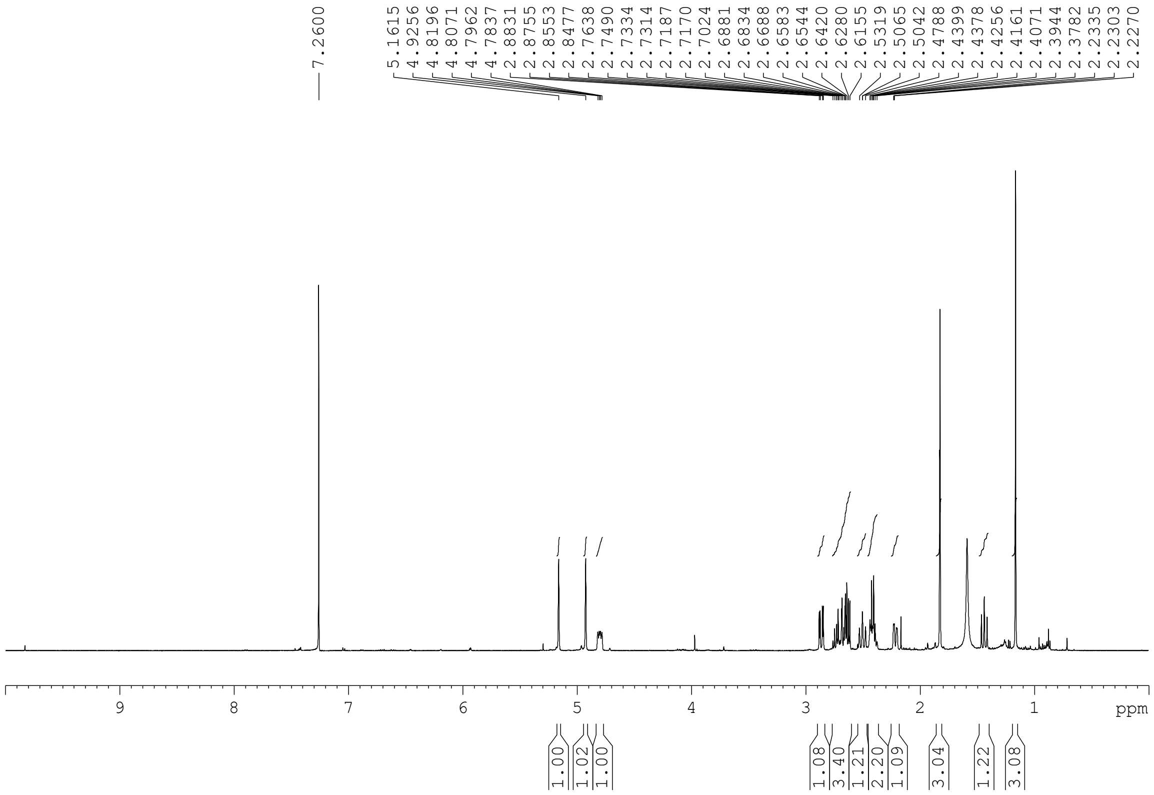

Figure S21 ^{1}H NMR spectrum of **3** (500 MHz, $CDCl_3$)

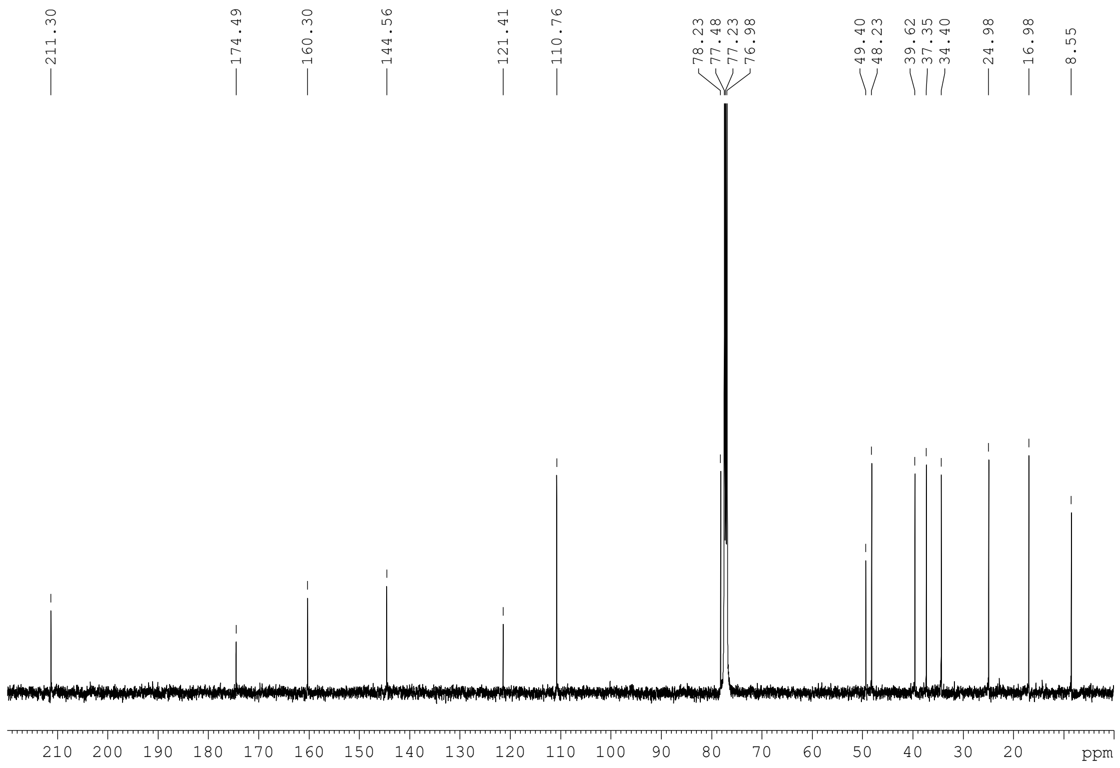

Figure S22 ^{13}C NMR spectrum of **3** (125 MHz, $CDCl_3$)

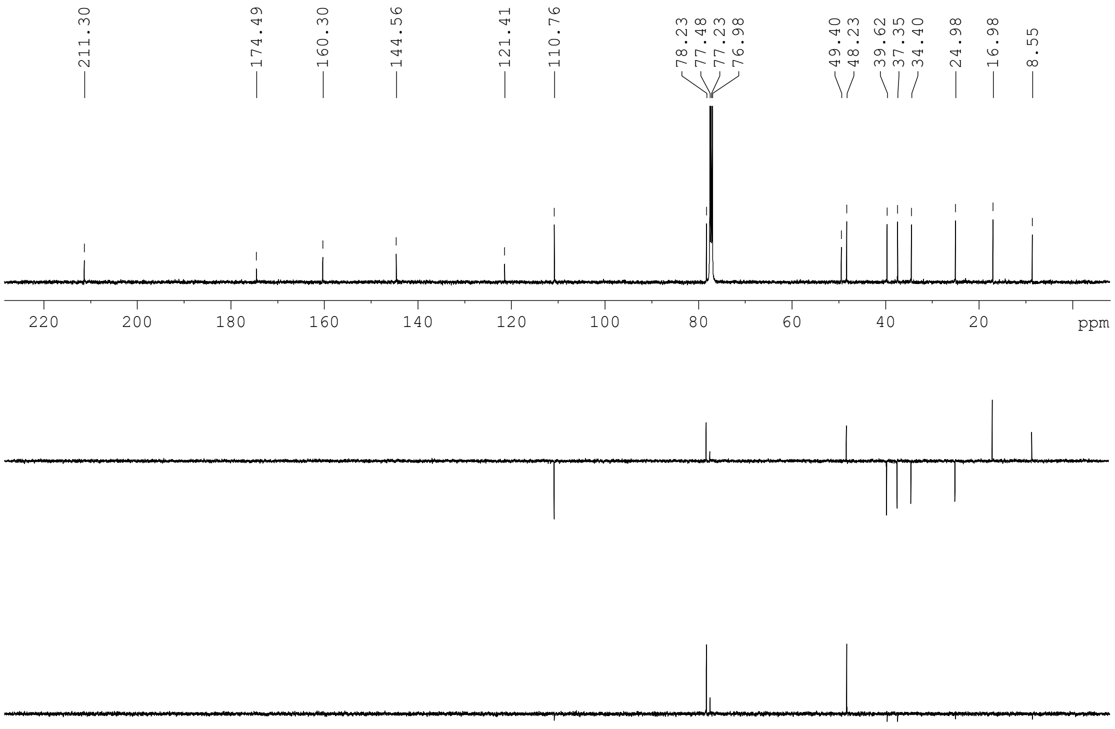

Figure S23 DPET spectrum of **3**

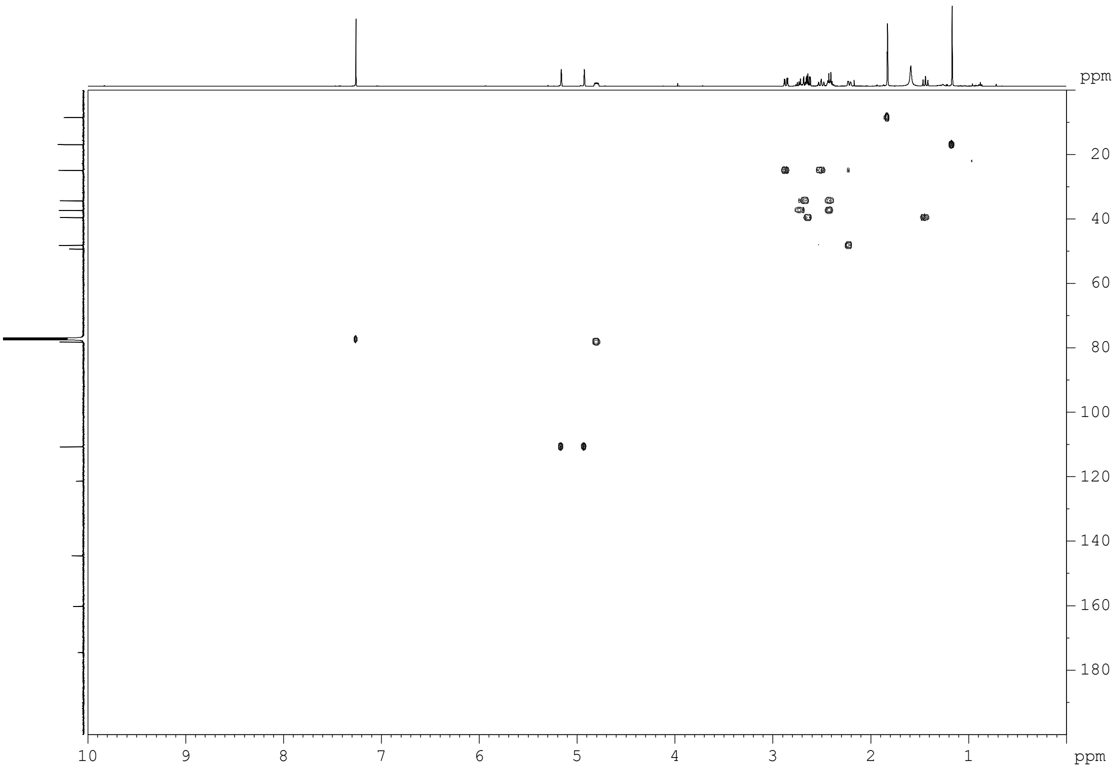

Figure S24 HSQC spectrum of **3**

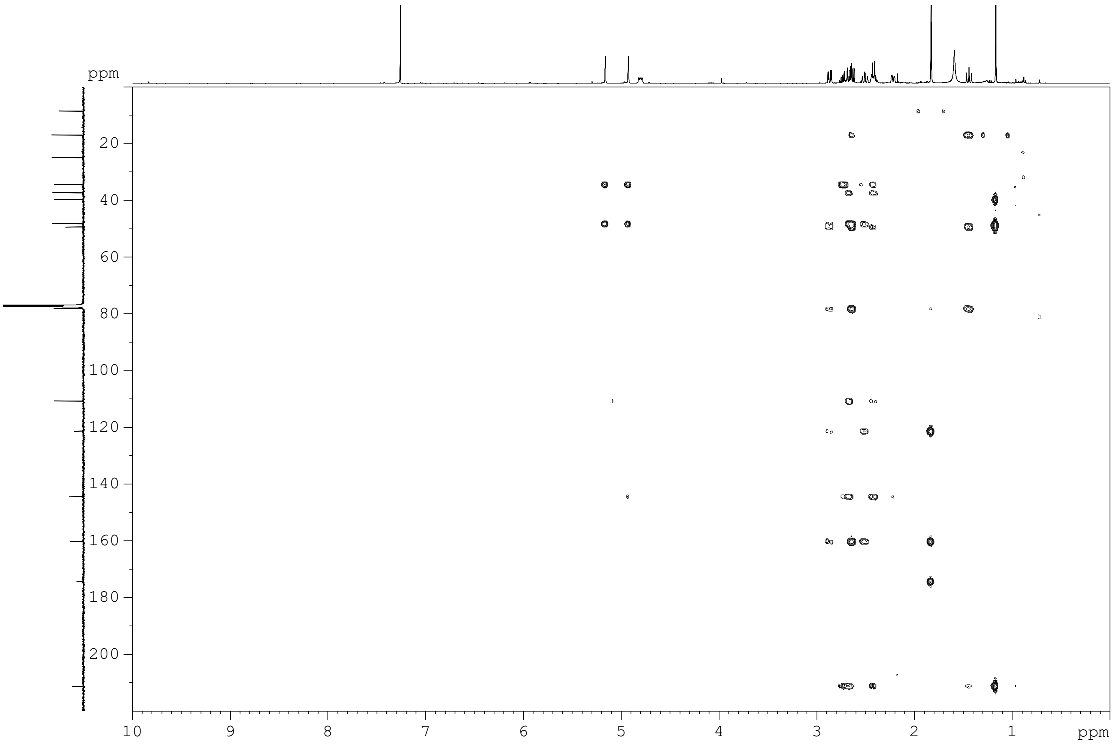

Figure S25 HMBC spectrum of **3**

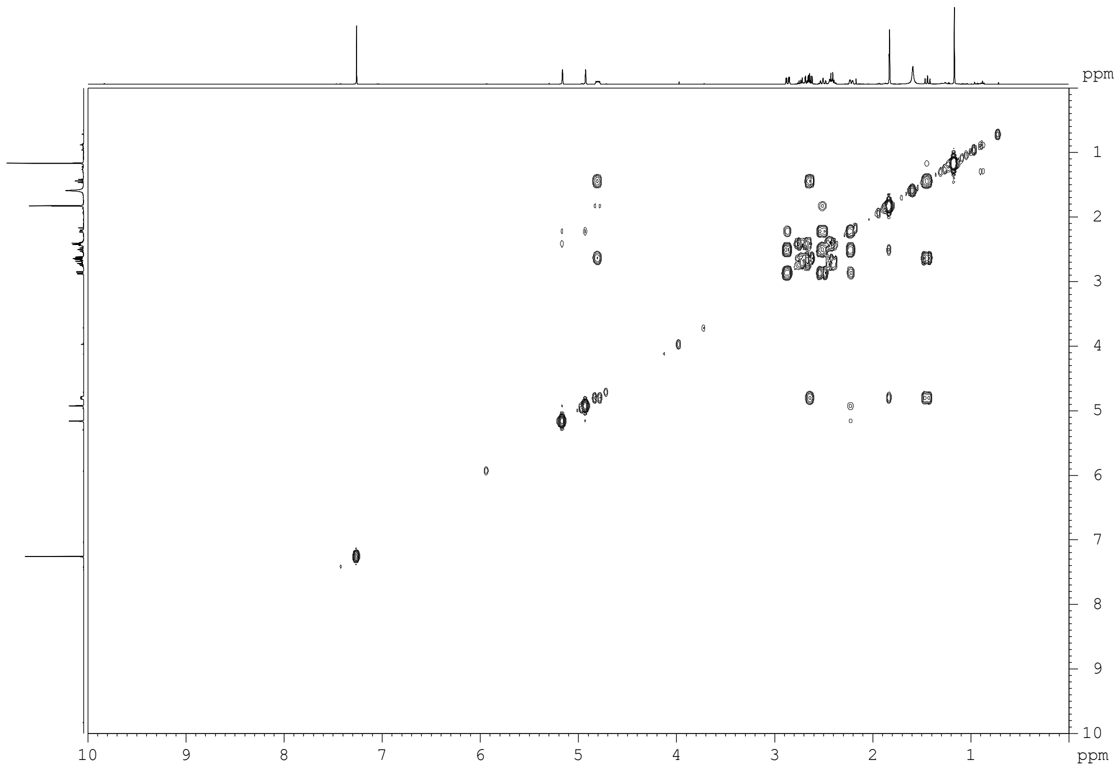

Figure S26 COSY spectrum of **3**

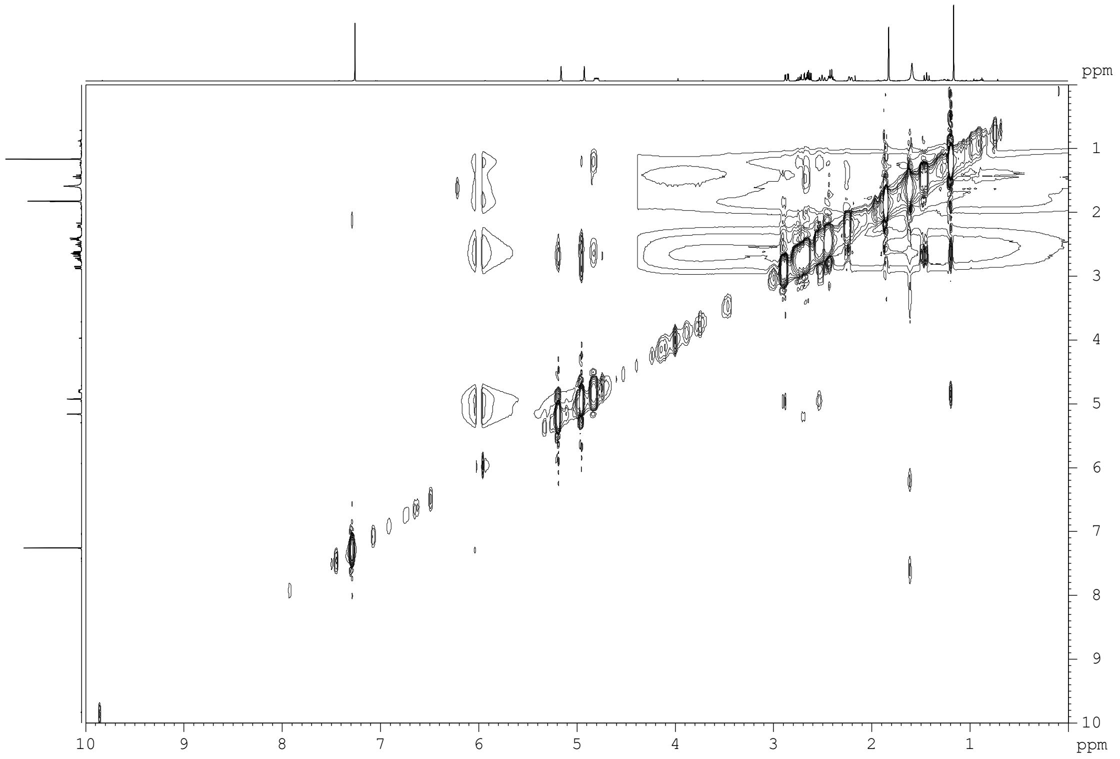

Figure S27 NOESY spectrum of **3**

Mass Spectrum SmartFormula Report

Analysis Info

Analysis Name	D:\Data\b9\D531110_000009.d	8/26/2016 3:53:44 PM
Method	broadband first signal	Operator: YU HSIAO-CHING
Sample Name	D5311-10	Instrument: BRUKER FT-MS solariX
Comment	ESI Positive	

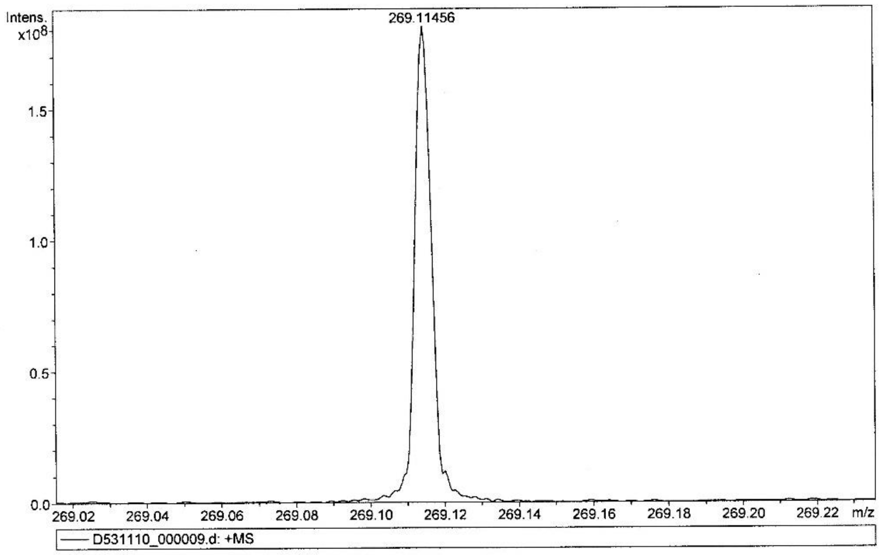

Meas. m/z	#	Formula	Score	m/z	err [mDa]	err [ppm]	mSigma	rdb	e⁻ Conf	N-Rule
269.11456	1	C 15 H 18 Na O 3	100.00	269.11482	0.25	0.94	3.9	6.5	even	ok

Figure S28 HRESIMS spectrum of **3**
